# Supplementary material for: An automated platform to monitor long-term behavior and healthspan in Caenorhabditis elegans under precise environmental control
Source: Commun Biol. 2020 Jun 10;3:297. doi: 10.1038/s42003-020-1013-2 (PMC7287092; doi:10.1038/s42003-020-1013-2)
Supplement: Supplementary file 1 — Supplementary Information [file 42003_2020_1013_MOESM1_ESM.pdf]

SUPPLEMENTAL MATERIALS

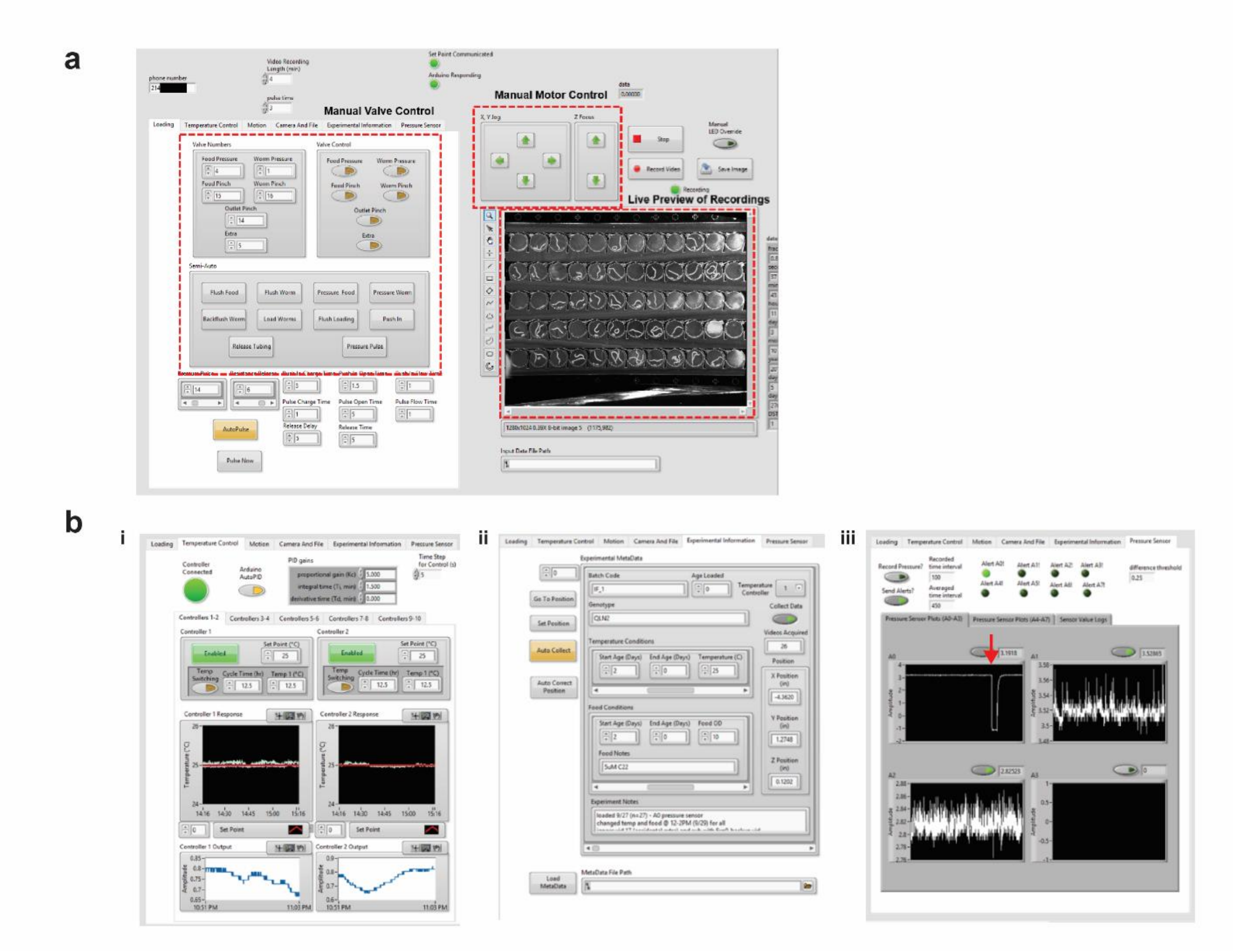

Supplementary Figure 1. HeALTH experimental GUI

a) Main page of the user GUI. This allows experimenters to manually control valves for fluid flow, move the camera and/or stage, and view/take records of devices in real-time. b) Additional subpanels of the GUI allowing for additional user control and input. i) Individual temperature control and monitoring for each detected temperature module. ii) Metadata and experimental information tracking for each device. iii) Continuous pressure monitoring and readout for automated clog detection. The drop in pressure (red arrow) indicates the opening of a downstream valve.

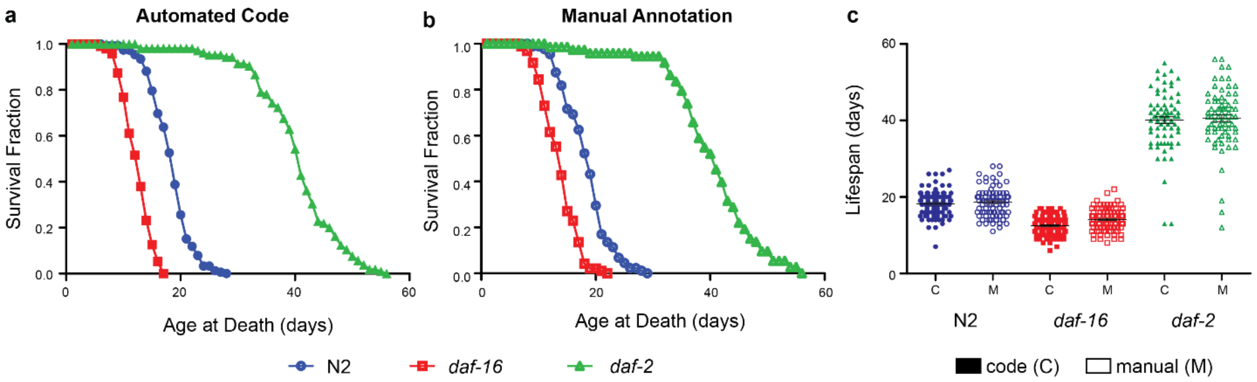

Supplementary Figure 2. Validation of automated live/dead code

a) Lifespan curves obtained from the automated analysis code for wild-type (18.20 days ± 0.37, n = 87 biologically independent animals), *daf-16* (12.48 days ± 0.26, n = 95 biologically independent animals), and *daf-2* (40.08 days ± 0.91, n = 73 biologically independent animals) populations. Error is reported as SEM. b) Manual annotation of death for the videos inputted into the automated analysis code for wild-type (18.48 days ± 0.42), *daf-16 (*13.78 days ± 0.32), and *daf-2* (40.29 days ± 0.94) populations. Error is reported as SEM. c) Comparison of average lifespans for wild-type, *daf-16*, and *daf-2* populations as found by the code and manual annotation. Error is reported as SEM. No significant difference was found between code outputted and manually annotated lifespans (log-rank test, *daf-16* p = 0.0162, N2 p = 0.3609, *daf-2* p = 0.6333).

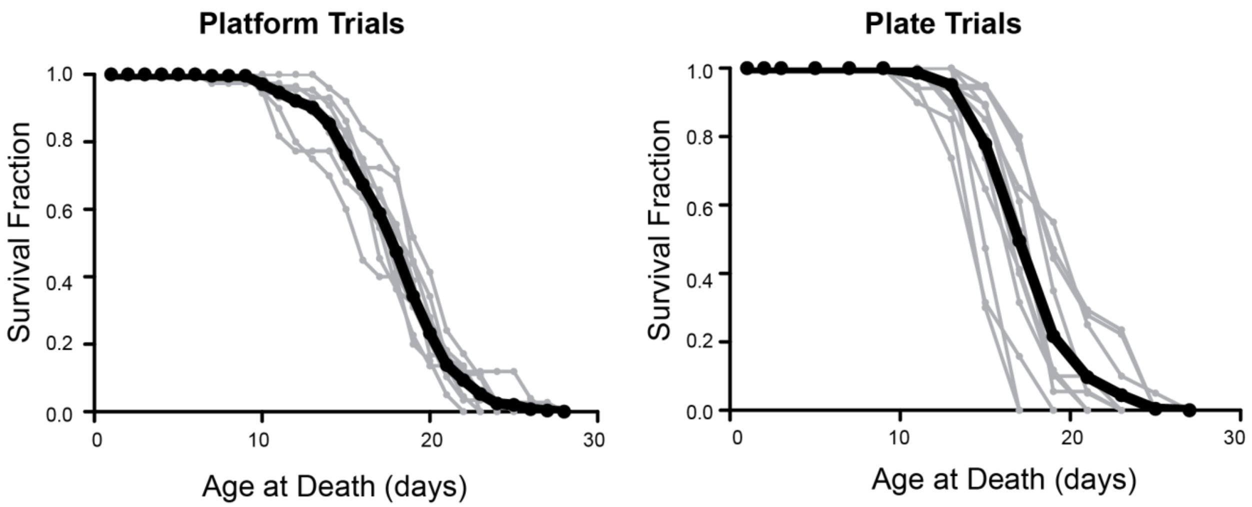

Supplementary Figure 3. Characterization of trial-to-trial variability

a) Lifespan curves for each trial for N2, cultured at 25°C with OD_{600}5 food concentration on the platform. Gray curves show individual trials while the black curve shows the aggregated lifespan curve. b) Lifespan curves for each trial for N2, cultured at 25°C with OD_{600}5 food concentration on plate. Gray curves show individual trials while the black curve shows the aggregated lifespan curve.

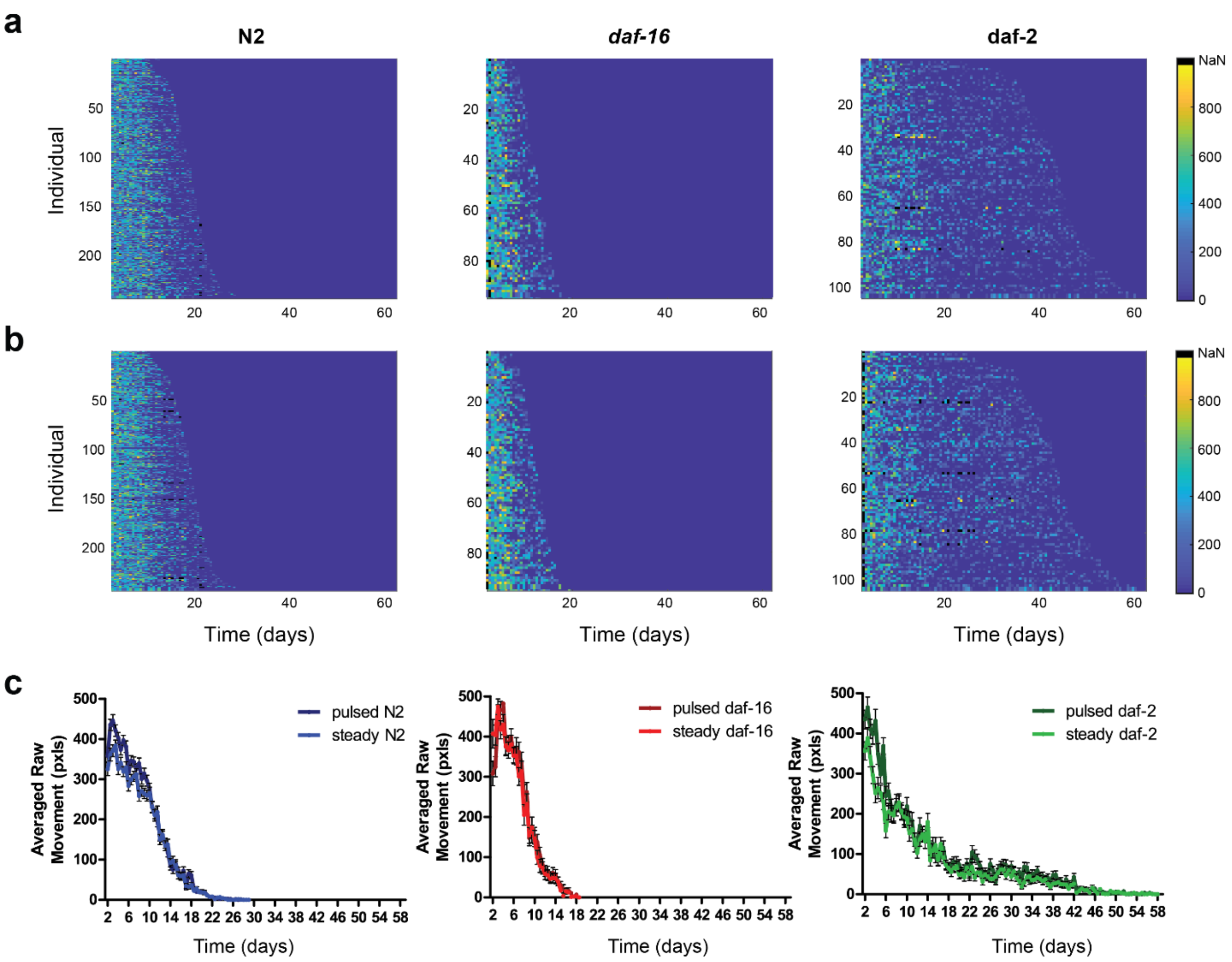

Supplementary Figure 4. Worms under constant fluid-flow demonstrated evoked behavior

a) Heatmaps of individual raw movement over time for N2, *daf-16,* and *daf-2* populations under constant flow conditions (~15µL/min). b) Heatmaps of raw movement over time for N2, *daf-16,* and *daf-2* populations after opening the downstream solenoid valve, creating a strong, pulse of fluid flow (~275µL/min) for 5 seconds. c) Population averages of raw movement over time for both the constant flow and 'pulsed' flow conditions across genotypes. No statistical significance was found between the two flow conditions (Kolmogorov-Smirnov test, N2 p = 0.9782, *daf-16* p = 1.000, *daf-2* p = 0.1413), indicating the constant flow conditions create enough of a mechanical stimulus to view stimulated behavior. Error bars are SEM.

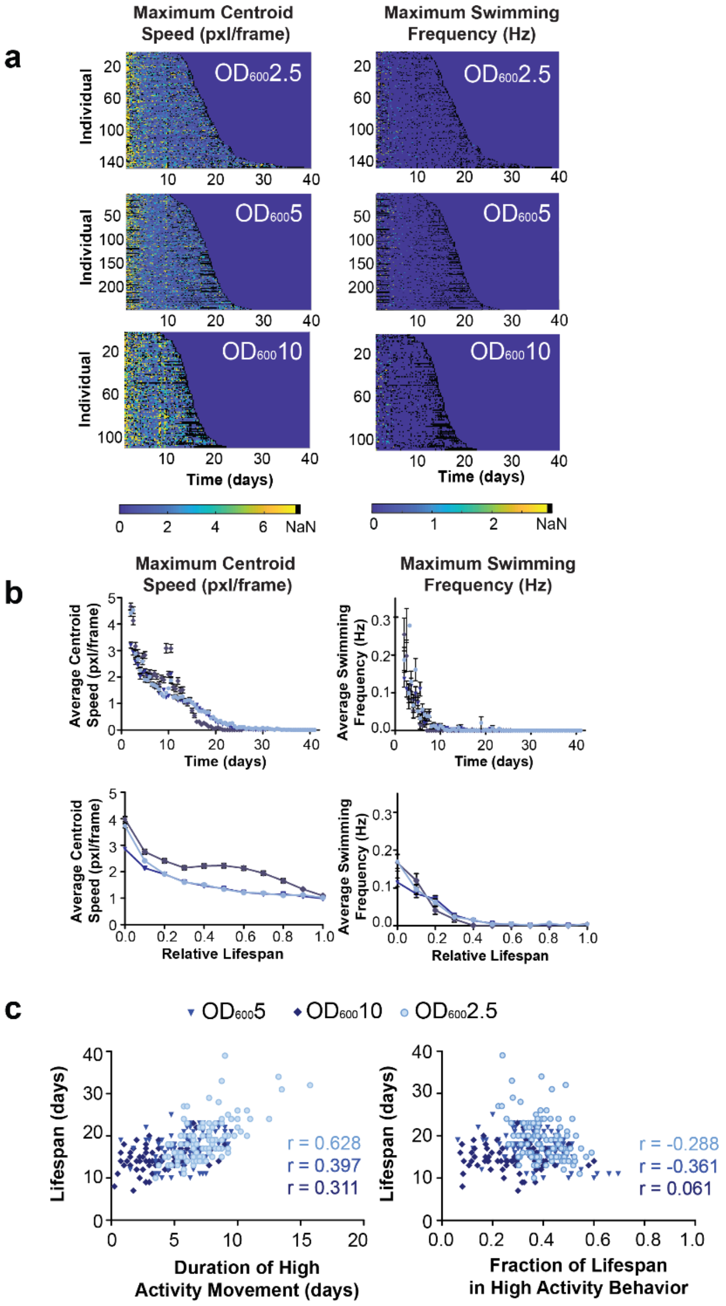

Supplementary Figure 5. Extended longevity and behavioral analysis for different food levels

a) Heatmaps of individual maximum centroid speed and maximum swimming frequency across OD_{600}2.5 (n = 147 biologically independent animals), OD_{600}5 (n = 245 biologically independent animals), and OD_{600}10 (n = 108 biologically independent animals) food levels over time. Individuals are N2 and cultured at 25°C. b) Population averaged activity and relative population averaged activity normalized to lifespan of maximum centroid speed and maximum swimming frequency across different food levels. Error bars are SEM. c) Scatter plots of duration of high activity for raw movement vs. individual lifespan and fraction of life with high activity for raw movement vs. individual lifespan across food levels. Pearson correlation coefficients are listed for each food level.

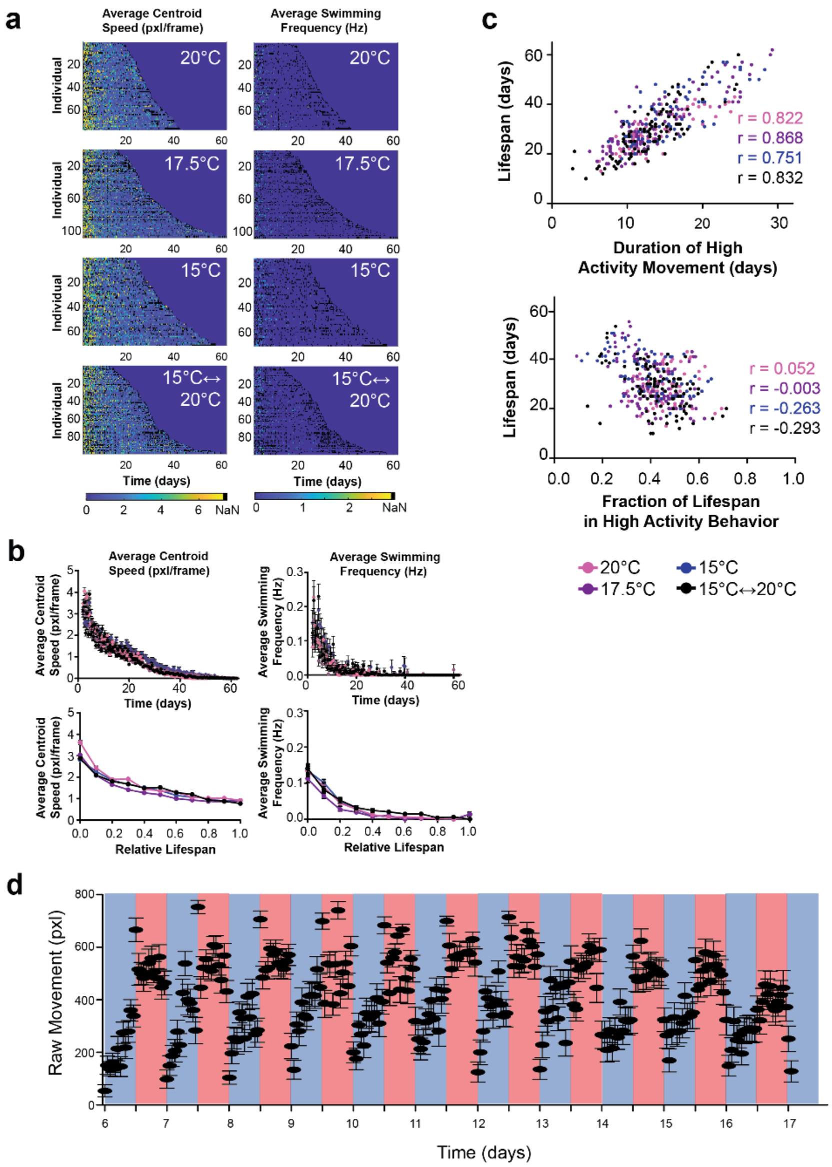

Supplementary Figure 6. Extended longevity and behavioral analysis for thermal perturbations

a) Heatmaps of individual maximum centroid speed and maximum swimming frequency at constant 20°C (n = 77 biologically independent animals), 17.5°C (n = 107 biologically independent animals), 15°C (n = 71 biologically independent animals), and oscillatory 15°C to 20°C (n = 99 biologically independent animals) thermal conditions over time. Individuals are N2 and cultured at food level $OD_{600}5$. b) Population averaged activity and relative population averaged activity normalized to lifespan of maximum centroid speed and maximum swimming frequency across different thermal conditions. Error bars are SEM. c) Scatter plots of duration of high activity for raw movement vs. individual lifespan and fraction of life with high activity for raw movement vs. individual lifespan across temperature conditions. Pearson correlation coefficients are listed for each thermal condition. d) Average raw movement of a population under oscillatory thermal swings (n = 26 biologically independent animals). Error bars are SEM. Blue background indicates a culture temperature of 15°C and a red background indicates a temperature of 20°C.

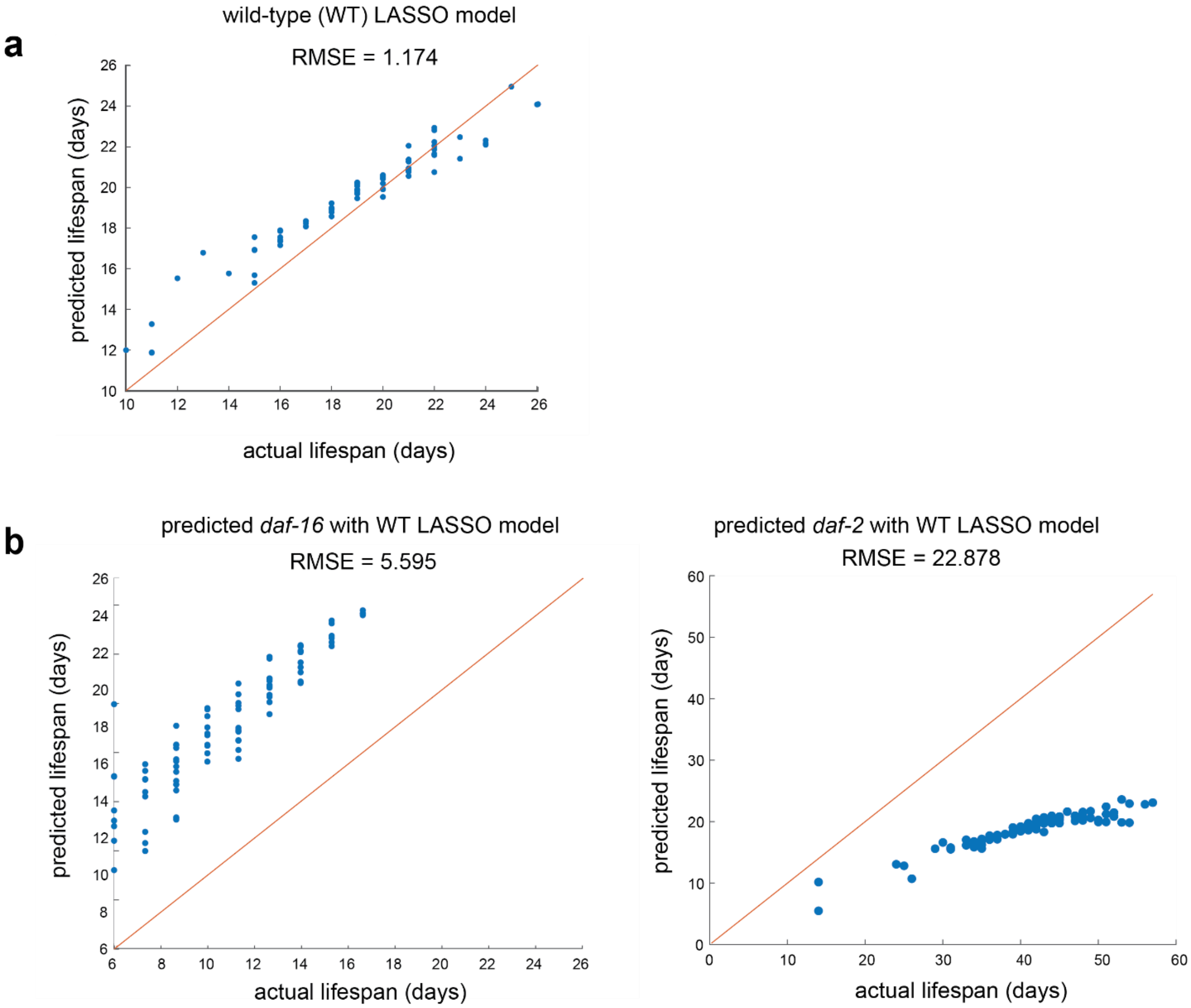

Supplementary Figure 7. Predictive models of lifespan based on behavioral decline

a) Comparing predicted lifespan v. actual lifespan across a wild-type population (n = 73 biologically independent animals) cultured at 25°C with $OD_{600}5$ using a LASSO regression model. Diagonal line indicates a theoretically perfect prediction. b) Comparing predicted lifespan v. actual lifespan for *daf-16* (n = 95 biologically independent animals) and *daf-2* populations (n = 105 biologically independent animals) using the corresponding wild-type predictive model (cultured at 25°C with $OD_{600}5$). The model consistently overpredicts the lifespan of *daf-16* individuals, while underpredicting the lifespan of *daf-2* individuals.

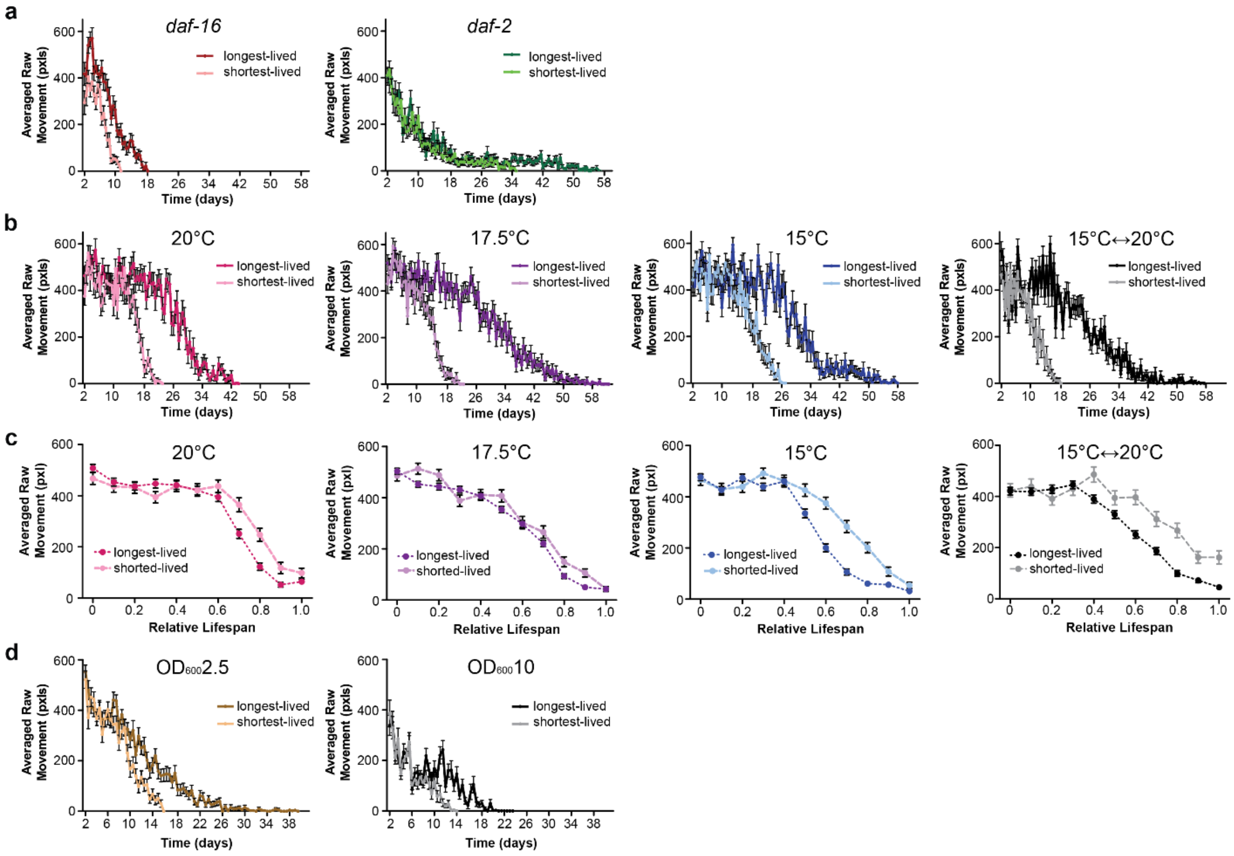

Supplementary Figure 8. Intrapopulation behavioral decline across genetic and environmental perturbations

a) Averaged raw movement over time of the short- and long-lived subpopulations at 25°C at OD_{600}5 food level across *daf-16* (shortest-lived: n = 23 biologically independent animals, longest-lived: n = 21 biologically independent animals) and *daf-2* (shortest-lived: n = 22 biologically independent animals, longest-lived: n = 23 biologically independent animals) populations. Populations are cultured at 25°C at food level OD_{600}5. Error bars are SEM. b) Averaged raw movement over time of the short- and long-lived wild-type subpopulations at OD_{600}5 food level across different thermal conditions. 20°C short- (n = 16 biologically independent animals) and long-lived (n = 16 biologically independent animals) subpopulations. 17.5°C short- (n = 24 biologically independent animals) and long-lived (n = 22 biologically independent animals) subpopulations. 15°C short- (n = 14 biologically independent animals) and long-lived (n = 15 biologically independent animals) subpopulations. 15°C<->20°C short- (n = 21 biologically independent animals) and long-lived (n = 22 biologically independent animals) subpopulations. Error bars are SEM. c) Corresponding average raw movement over normalized, relative lifespan of the short- and long-lived subpopulations across different thermal conditions. Error bars are SEM. d) Averaged raw movement over time of the short- and long-lived wild-type subpopulations at 25°C across OD_{600}2.5 (shortest-lived: n = 45 biologically independent animals, longest-lived: n = 37 biologically independent animals) and OD_{600}10 (shortest-lived: n = 22 biologically independent animals, longest-lived: n = 26 biologically independent animals) food levels. Error bars are SEM.

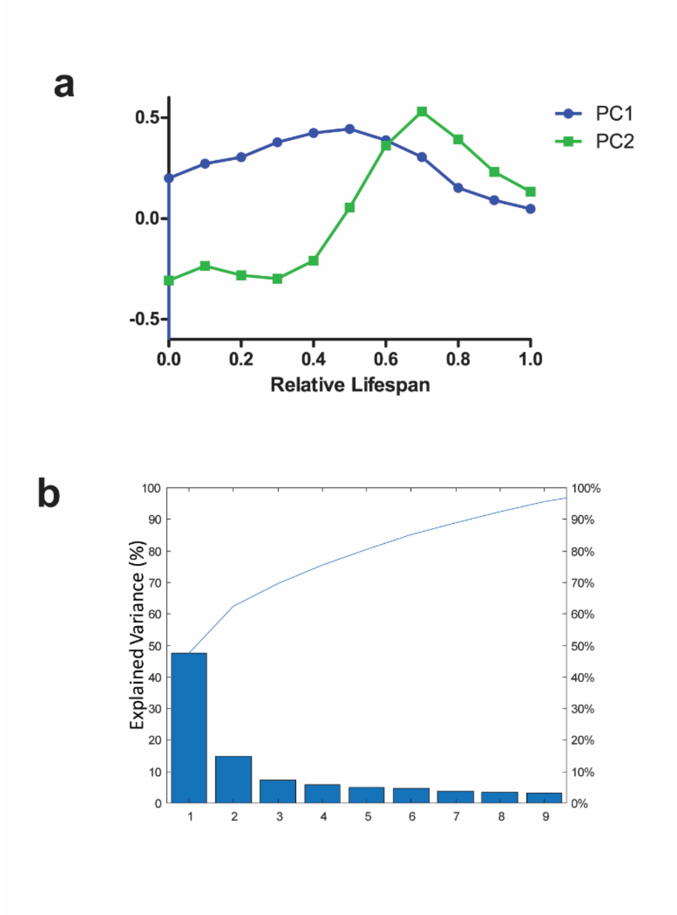

Supplementary Figure 9. Characterization of PCA

a) First two principle component curves. b) Scree plot of explained variance with each principle component.

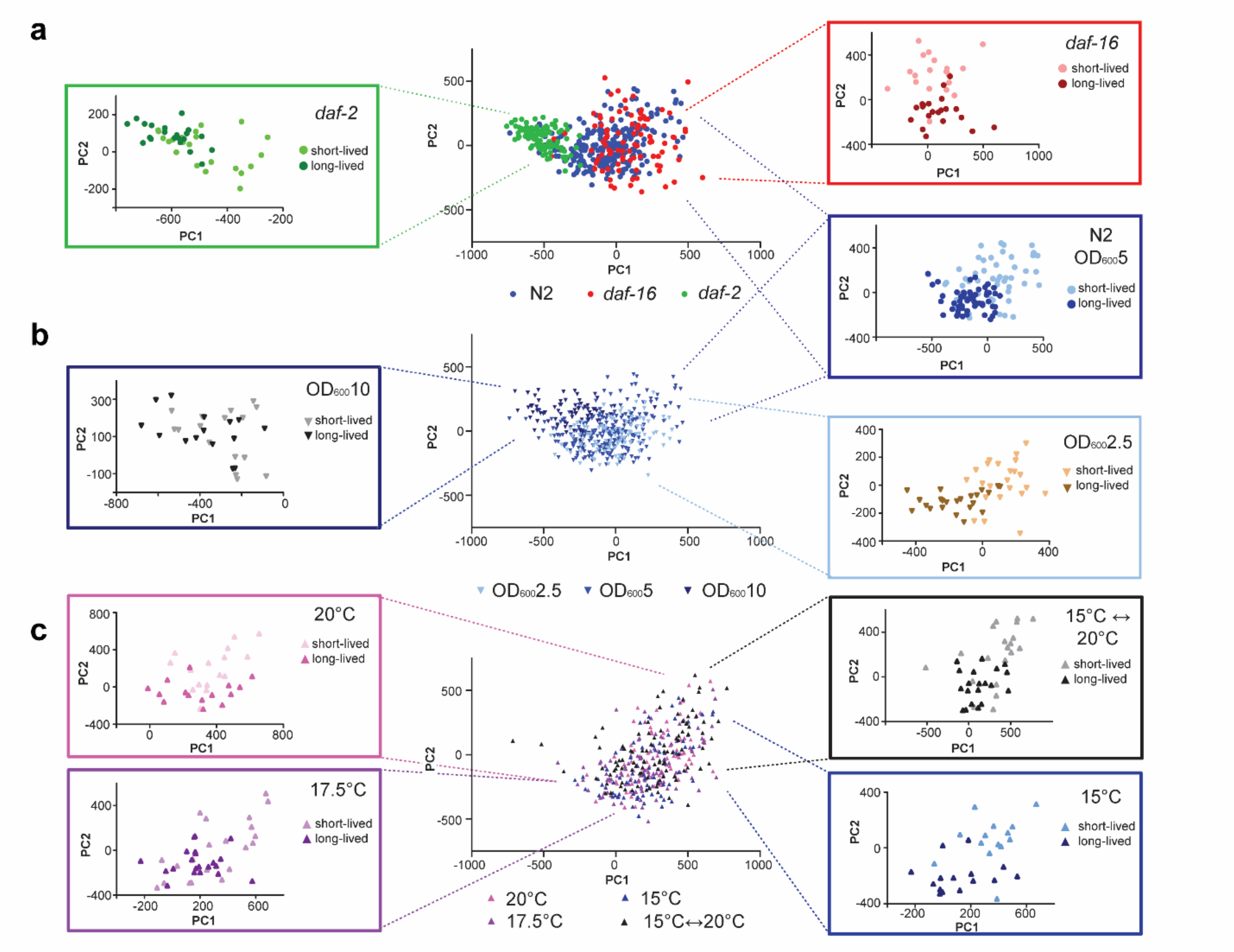

Supplementary Figure 10. Intrapopulation variation within the PC space

a) Individuals cultured at 25°C at food level $OD_{600}5$. Inserts show the spatial location and division of the short-lived and long-lived cohorts across the different genotypes. b) Wild-type individuals cultured at 25°C. Inserts show the spatial location and division of the short-lived and long-lived cohorts across the different food levels. c) Wild-type individuals cultured at food level $OD_{600}5$. Inserts show the spatial location and division of the short-lived and long-lived cohorts cultured at different thermal conditions.

	PLATFORM VARIABILITY		PLATE CONTROL VARIABILITY	
Source of Variability	Variance	Percent of Variance (%)	Variance	Percent of Variance (%)
Trial	0.8281	6.41	2.2211	27.16
Trial x Plate	0.1568	1.21	0.0237	0.29
Individual Variation	11.9275	92.26	5.9189	72.38
Total	**12.9124**		**8.1773**	

Supplementary Table 1. Sources of variability across experimental trials

Variance components and the percent of variance due to difference sources of variability across trials cultured on the platform and on plate. Variance was calculated using a GLM mixed-effect model [1]. Total variation across the two culture methods is comparable. Variance attributed to different trials is lower within the platform. The individual variation is higher; however, this could be due to bacteria. A single trial of the plate assay was seeded with the same batch of bacteria, while multiple batches were grown and used on the platform due to the high consumption rate of bacteria culture. As a result, the high ‘individual variation’ could be due to variation across bacterial cultures.

Supplementary Note 1. HeALTH Design Documentation

We present HeALTH, a platform for performing automated longitudinal healthspan studies for *C. elegans* under precise environmental control. The following documentation is a brief overview on how the entire platform was built and customized for the set of experiments shown in the article. By design our system is modular and scalable. As a result, it can be customized depending on the requirements of the user. The complete bill of materials (BOM) is organized by subsystem and is listed at the end of the documentation. Details for the specific parts mentioned in the text can be found in the BOM and are referenced by item number within each system sub-section. All CAD files are uploaded to GrabCAD at: https://workbench.grabcad.com/workbench/projects/gcwDW30oR56MuBI2OsZ-ge4yfAhwFzatnB6-7xhbRppU0R#/space/gcSlzhGMjRykJLl3yZbV99GF6oXy3TtZqJsoVO-CstAUnH

1. Imaging System

The imaging system consists of a single camera mounted on an automated x-y stage that travels and record the behavior of populations across different devices on the platform. To create a motorized stage system with a wide range of motion and precise X, Y, and Z directional movement in a simple, efficient manner we modified a commercially available DIY CNC stage kit, where three separate stepper motors driven by a microcontroller are coupled to threaded rods drive motion across the three directions. We chose the MyDIYCNC Desktop CNC Machine from MyDIYCNC. Unfortunately, the product and company now no longer exist; however, the general idea and modifications we made should be translatable to other, currently available DIY stage kits. As a result, the exact dimensions and modifications should be modified as needed to fit the user's actual system. For reference, our system has an X range of motion of ~15", Y motion of ~13", and Z motion of ~3", with a resolution of 10 µm across all directions. The following sections detail how to modify the motorized stage and how to create the imaging and illumination system.

1.1 Modifying the motorized stage

One of the major modifications necessary for many CNC stage kits is to alter the height of the machine in order to allow for the imaging components. To do so, we added height extension panels (#18) to the sides of the assembled CNC stage. Additionally, to make the system more compatible with fluids, we altered the stage material from patterned metal sheet to acrylic (15"L x 13"W x 3/16", #19). We also removed the router mount and replaced it with a custom machined HDPE part that holds and connects the lens clamp (#4). Based on the type of kit purchased, the screw holes will need to be adjusted; however, the holder for the clamp should be constant. See below for an image of the imaging system.

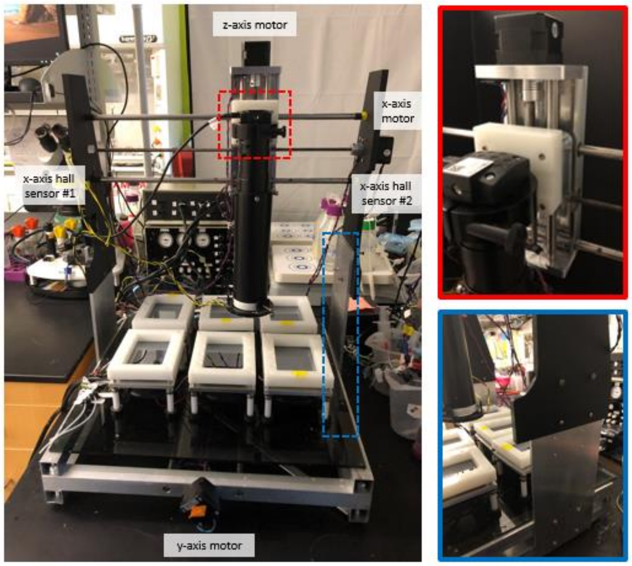

Overview of the imaging system. (*left*) Image of imaging system with temperature modules on the stage. Major components of the motorized stage are labeled. (*top right, red insert*) close-up of the machined HDPE lens clamp holder. *(bottom right*, blue insert) close-up of the machined height extension panels

To more easily integrate motor control with the other platform components we rerouted the motor wiring (#16-17) to a small microcontroller (#23, #26-28), which then communicates with the integrated LabVIEW GUI. The microcontroller outputs digital pulses to drive motion from the stepper motors. We also added hall effect proximity sensors (#20) on the edges of each axis for positional information and connected it to the microcontroller as a limit switch control. The x-axis halls sensor locations are labeled in the image shown above. The motor circuit is housed in a custom machined project box (6.112" L x 4.612" W X 2.356", #22). The system is powered by a 12V power supply (#24-25). See below for the electronic circuit diagram, illustrating the connections for a single motor and its corresponding sensors.

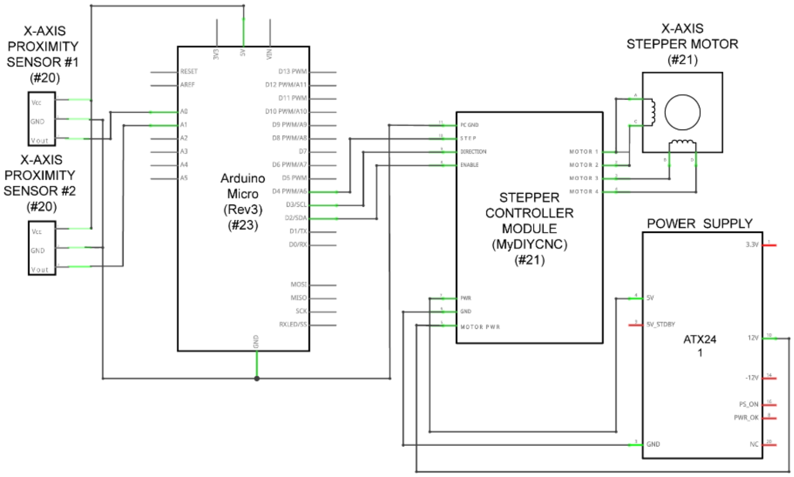

Circuit diagram of the motor drivers. This is a representative wiring diagram illustrating the connections for a single axis (the x-axis). The motors and proximity sensors for the other axes can be added via separate stepper controller modules and connected to the open analog input pins (for the sensors) and digital output pins (for the motor controllers).

1.2 <u>Creating the imaging and illumination system</u>

To record the populations, we utilize a low-cost CMOS camera (#1) coupled with a 10X zoom lens (#2) to modify the camera’s magnification and field of view in order to observe an entire device. The lens is then mounted on the HDPE mount (#3). To increase video contrast, we machined a collimator (#5) that is tension mounted with screws on the end of the zoom lens. To reduce the amount of light exposure on

organisms, we have an automated LED illumination source that automatically switches on when recording behavioral videos or by control of the experimenter via the GUI. The LED source is comprised of two concentric red LED rings (#6-7). The LED rings are mounted on a custom made, laser-cut acrylic LED holder (#8) and is tension mounted via screws on the end of the collimator. See below for an image of the imaging and illumination system. The LEDs are wired to plug connectors (#16-17) and are controlled by a microcontroller connected to computer USB port (#10-11, 15). The system is powered via a 12V power adapter (#12-14) and is contained within a custom machined project box (4.724" L x 3.157" W X 2.311", #9).

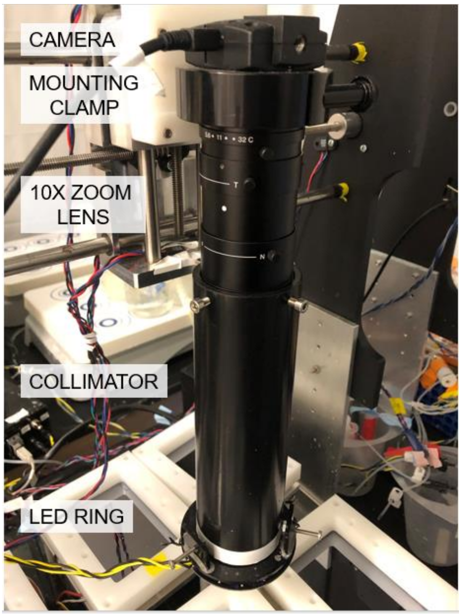

Close up view of the imaging components.
Major components are labeled.

2. Temperature Control System

Temperature plays a significant role in the development and aging process of *C. elegans*. As a result, it is crucial to provide and maintain precise thermal control over the population. To enable modularity and flexibility for different thermal conditions we developed small thermal modules that can hold up to 4 microfluidic devices. The circuit to control the thermal modules is modular, with a single circuit corresponding to a single thermal module. As a result, it is easy to scale the number of modules according to the experimental need. The BOM assumes the creation of 6 temperature modules, which is the number of modules that can comfortably fit on the platform. However, based on the size of the platform, that can be easily scaled. The following sections detail how to build a single thermal module, along with its corresponding circuitry.

2.1 Building the thermal module

To provide precise heating and cooling capabilities, we used Peltier plates (#24) embedded within machined thermal modules. The module is composed of several layers. A thin copper plate (4.25”L x 3”W x 0.125”, #27) is in thermal contact with a Peltier module, allowing for consistent thermal distribution across a larger surface. To allow for dark-field imaging via reflected light from the over LED ring illumination, a reflective silicon surface (#37) is layered above the copper plate. The Peltier is insulated by a layer of foam (#30) to reduce unintended thermal loss. To prevent overheating and for improved heat dissipation the opposite side of the Peltier is flush with an aluminum plate (5.25”L x 4”W x 0.25”, #29). The plate is attached to a copper heat sink and cooling fan (#2, 25). The Peltier, thermocouple, and cooling fan wiring extend and connect to the main temperature controller via plug connectors (#16, 23). The layers are compression mounted together via screws (#36), which are coupled with machined plastic coverings (#32) and rubber feet (#33) to support and stabilize each module on the platform surface. A machined plastic outer casing (#28) aids in the compression and allows for long-term mounting of microfluidic devices on the module via screwed clamps (#31, 34-35). A thin layer of thermal paste (#38) is between all layers of the module for improved heat transfer. The CAD files for all machined parts can be found on GrabCAD. See below for images of the thermal module and of microfluidic devices mounted on the module.

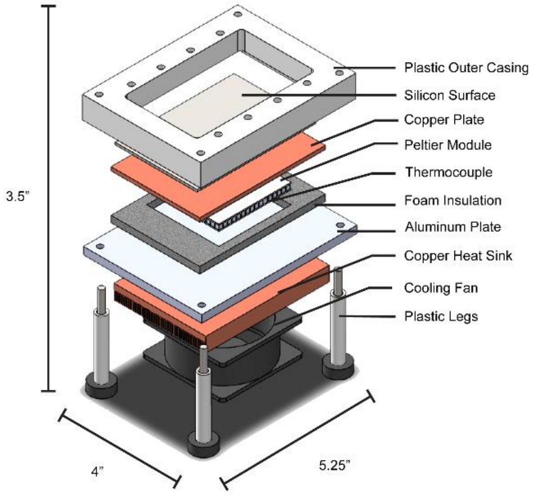

Exploded view of the thermal module.

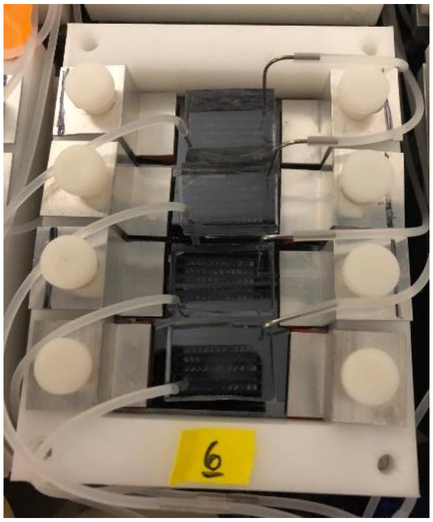

Microfluidic devices clamped onto the thermal module. The plastic outer casing provides threads for the aluminum machined clamps to be screwed into, providing pressure to clamp the devices in place over long periods of time.

2.2 Creation the temperature control circuit

The temperature control circuit needs to be able to read analog temperature values from the thermocouple embedded within the temperature module and adjust outputted voltages to account for changes in the set value or ambient temperatures. To do so, we use a basic voltage divider circuit to read resistance changes in the thermistor. The values are reported to the microcontroller, connected to a computer via USB port, and converted via a look-up table into temperature values (#15, 20-21). The required output is then calculated via PID controller, which alters the output magnitude (via PWM analog output) and direction (via digital output) using a DPDT relay, thus allowing heating and/or cooling to reach the desired set point. Due to the high current requirements of the Peltier, we incorporated a voltage buffering state after the PWM output (#3-10). The circuit diagram is shown below and is soldered onto prototyping breadboards (#12). Each circuit was wired to the thermal module via plug connectors (#16-17, 23). The entire system is powered by an external power supply (#18-19,22). All breadboards are contained and fastened (#12) within a custom machined project box (8"L x 12"W X 4 ", #1). For added heat dissipation of the circuits, an array of fans (#2) are embedded within the sides of the enclosure, shown below.

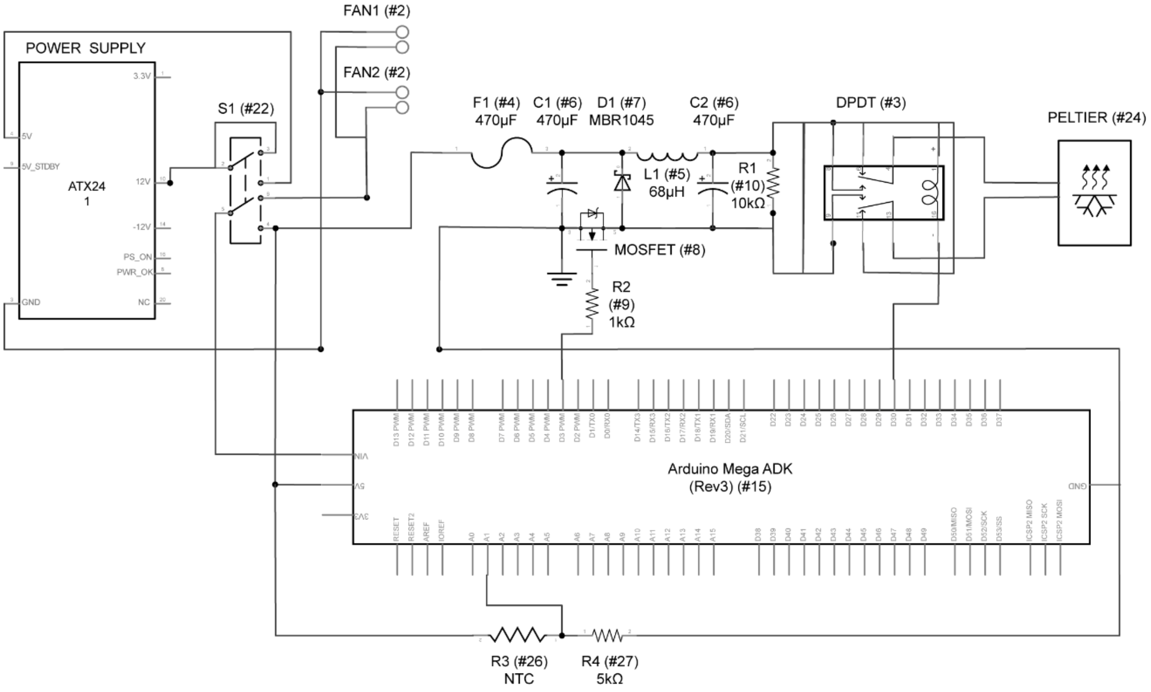

Circuit diagram for the temperature control circuit. This is a representative example of a single temperature controller module. It can be easily adapted for more controllers by increasing the number of modular circuits and thermal modules connected to the power supply and microcontroller.

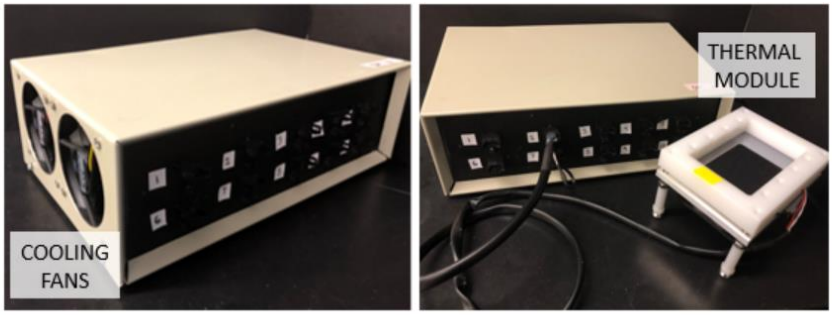

Temperature Controller System. (*left*) Temperature control box with cooling side fans. (*right*) Control box with thermal module

3. Fluid Flow System

Maintaining a constant food level across the entirety of the worm's lifespan and delivering a wide range of food levels on-chip is largely depending on being able to ensure a constant fluid flow over time to maintain desired food levels within the device. As a result, there is a need to 1) deliver a consistent driving pressure over time for pressure-based fluid flow, and 2) reduce bacterial accumulation and clogging within the system to ensure constant flow. To do so, we have developed two different systems to ensure robust fluid flow and culture over long periods of time.

3.1 Pressure Delivery System

To ensure automated food delivery and we must be able to set and deliver a consistent pressure value over long period of time. Furthermore, to automatically load the microfluidic devices (the process of which is detailed in prior work [2]), it is necessary to quickly apply and release a variety of pressures and activate off-chip solenoid pinch valves to start and stop flow on-chip. To accomplish these needs, we developed a pressure delivery system which acts as an integrated pressure controller system to incorporate independent pressure controls, pneumatic valves, and accessory outlets to drive components such as solenoid pinch valves to control flow off-chip. See below for an image of the system.

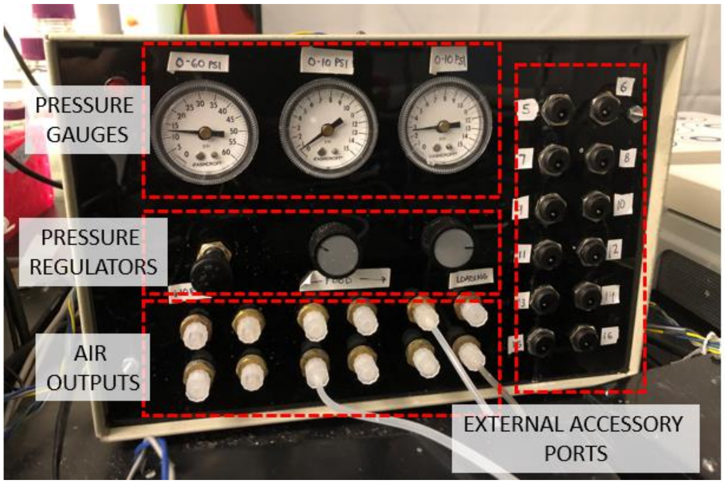

Pressure delivery system. User controls (ex. pressure regulators) are highlighted and labeled.

Pressure from an in-house air source is inputted to the system and split across three sets of pressure regulators, with ranges from 0-15PSI to 0-60PSI (#9, 12-14, 16, 18-20). The set pressures are easily modulated and the values are indicated via pressure gauges (#10-11). Two of the regulators are attached to pneumatic valves, allowing electronic switching between the regulated input value and atmospheric value for rapid pressurization/depressurization (#1-3). One directly connects the pressure output to the regulated input for constant pressure-flow applications. Outputted air flow is connected to pressurized reservoirs via tubing with luer connectors (#15, 17, 21). To easily control the valves and accessory power outputs, they are connected to a USB-controllable LED controller, which controls of all electronic outputs to a single USB connection to a computer (#5-8). It is powered via an external power supply (#22, 24, 26), and has panel mounts for external accessory components, such as solenoid pinch valves (#23, 25, 27-29). The parts are housed in a custom-machined project box (11.5"L x 7.5"W x 7"H, #4, 30). Shown below are diagrams of the air flow the electronic circuit diagram.

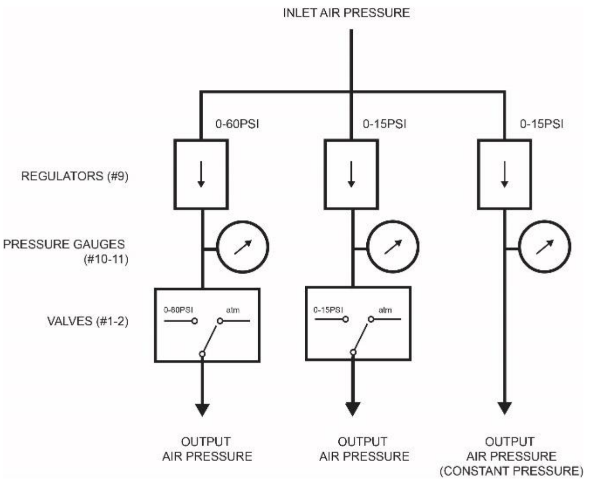

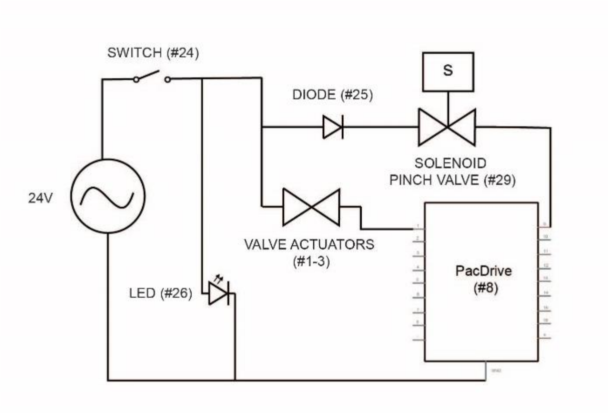

Pneumatic diagram of the pressure delivery system

Circuit diagram for the pressure box. This is a representative circuit showing the use of one actuating valve and one solenoid pinch valve. More can be easily added in parallel and connected to separate pins in the PacDrive controller.

3.2 Clog Detection System

Even with in-line filters and constant bacterial agitation within the reservoir via stir bar, high food concentrations can create bacterial accumulation in the upstream tubing and filter. This impacts the flow rate within the device and can lead to clogging. To prevent that, we engineered a continuous pressure monitoring device that is able to sense significant pressure drops which signal the presence of clogging upstream. The system then notifies the experimenter via text so that certain parts, such as filters, can be changed before the formation of clogs.

Miniature pressure sensors (#4) are wired as part of an amplified Wheatstone bridge circuit (#5-7). Changes in voltage are read by a microcontroller (#1) and reported in the LabVIEW GUI. The sensors are connected to the system via plug connectors (#2-3) and are powered by an external 12V supply (#8-9). The circuit is soldered to a prototyping breadboard (#10). It is housed in the same project-box as the pressure delivery system. See below for the electronic circuit diagram.

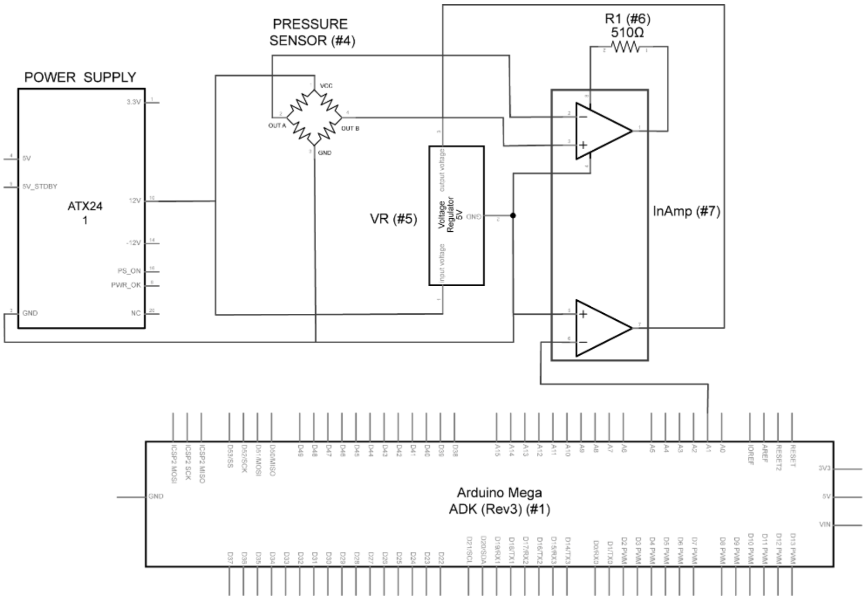

Circuit diagram of the clog detection system. This is a representative circuit showing the connections for a single sensor. More sensors can be added and connected to different analog input pins of the microcontroller.

HeALTH Bill of Materials

Below is a complete list of materials used to create HeALTH. This list assumes the creation of 6 temperature modules, which is the number of modules that can comfortably fit on the platform, along with 6 pressure sensors and solenoid valves, for each set of devices on a thermal module. Items are organized by subsystem and are numbered accordingly within the section. The prices and items are current as of June 2018; however, availability and pricing could be subject to change. Notes, such as whether the part needs to be machined, are included as well.

	Item #	Item Name/Description	Vendor	Part Number/ID	Number	Unit Price	Total Cost	Notes
Imaging System	1	DCC1545M - USB 2.0 CMOS Camera	ThorLabs	DCC1545M	1	376.62	376.62	
	2	10X (13 - 130mm FL) C-Mount, Close Focus Zoom lens	Edmund Optics	#54-363	1	595	595	
	3	10X Zoom Lens Mounting Clamp	Edmund Optics	#56-547	1	60	60	
	4	HDPE mount (connecting lens mounting clamp to the CNC system)	McMaster Carr	8619K613	1	11.05	11.05	needs to be machined (see CAD file)
	5	Acetal Resin Tube collimator	McMaster Carr	1830T289	1	15.32	15.32	needs to be machined (see CAD file)
	6	Red LED Ring (60mm Diameter)	SuperBrightLEDs	AE60-R30-BK	1	7.95	7.95	
	7	Red LED Halo Ring (80mm Diameter)	SuperBrightLEDs	AE80-R48-BK	1	11.95	11.95	

	8	Acrylic LED Ring Mount (connecting LED rings to collimator)	McMaster Carr	8505K741	1	7.14	7.14	needs to be machined (see CAD file)
	9	Project Box for LED Control (4.724" L x 3.157" W (119.99mm x 80.19mm) X 2.311" (58.70mm))	Digikey	HM110-ND	1	6.5	6.5	needs to be machined
	10	PacDrive USB driver board Type: Special (Outputs OFF at power on)	Ultimarc	PPACDSP+L U04	1	30	30	
	11	USB 2.0 Cable (A Male to B Male)	Digikey	1175-1088-ND	2	3.25	6.5	
	12	Toggle Switch (SPDT)	Digikey	EG2350-ND	1	2.43	2.43	
	13	12V AC/DC Wall Power Adapter	Digikey	62-1230-ND	1	12.21	12.21	
	14	Power Barrel Connector Jack	Digikey	EJ508A-ND	1	1.38	1.38	
	15	USB Type B - Type A Adapter	Allied Electronics	70126196	2	10.05	20.1	
	16	4 wire plug connector pin (male)	Digikey	3M155973-ND	7	3.22	22.54	
	17	4 wire plug connector pin (female)	Digikey	3M156151-ND	7	3.5	24.5	
	18	Aluminum platform sides	McMaster Carr	8975K432	1	23.18	23.18	needs to be machined (see CAD file)
	19	Acrylic Platform	McMaster Carr	8505K731	1	33.13	33.13	needs to be machined (see CAD file)
	20	Hall Effect Magnetic Sensors	Mouser	934-551003H02A	6	6.88	41.28	
	21	DIY CNC kit	MyDIYCNC	MyDIYCNC Desktop CNC Machine	1	445	445	Exact system is currently discontinued, but should be able to adapt to other, common DIY CNC kits
	22	Project Box for Motor Control (6.112" L x 4.612" W (155.24mm x 117.14mm) X 2.356" (59.84mm))	Digikey	377-1218-ND	1	9.7	9.7	needs to be machined
	23	Arduino Micro	Arduino	A000053	1	19.8	19.8	
	24	3 wire plug connector pin (male)	Digikey	3M155968-ND	1	2.72	2.72	

	25	3 wire plug connector pin (female)	Digikey	3M155845-ND	1	2.92	2.92	
	26	4 position terminal block for attaching wire to board	Digikey	A98335-ND	4	2.68	10.72	
	27	2 position terminal block for attaching wire to board	Digikey	A98333-ND	2	0.88	1.76	
	28	Solder prototyping breadboard	Digikey	SBB400-ND	1	3.99	3.99	
**Temperature Control System**	1	Project Box for Temperature Control (8.000" L x 12.000" W (203.20mm x 304.80mm) X 4.000" (101.60mm))	Digikey	HM302-ND	1	60.5	60.5	needs to be machined
	2	CPU Cooling Fan	Amazon	R4-S8R-20AK-GP	10	5.71	57.1	
	3	DPDT relay	Digikey	PB383-ND	6	2.36	14.16	
	4	On-board 4A fuse	Digikey	F2344CT-ND	6	1.842	11.052	
	5	68 uH inductor	Digikey	AIAP-03-680K-ND	6	1.55	9.3	
	6	470 uF capacitor	Digikey	P16299-ND	12	0.941	11.292	
	7	Schottky diode	Digikey	MBR1045PBF-ND	6	1.1	6.6	
	8	logic level MOSFET	Digikey	2SK4017Q-ND	6	0.67	4.02	
	9	1 kohm resistor	Digikey	PPC1.0KBCT-ND	6	0.44	2.64	
	10	10 kohm resistor	Digikey	PPC10KBCT-ND	6	0.44	2.64	
	11	Solder Prototyping Breadboard	Digikey	SBB206-ND	6	2.49	14.94	
	12	Breadboard Adhesive Fastener	Digikey	RPC1096-ND	12	0.932	11.184	
	13	2 position terminal block for attaching wire to board	Digikey	A98333-ND	6	0.88	5.28	
	14	4 position terminal block for attaching wire to board	Digikey	A98335-ND	6	2.68	16.08	
	15	Arduino MEGA	Arduino	A000067	1	38.5	38.5	
	16	4 wire plug connector pin (male)	Digikey	3M155973-ND	6	3.22	19.32	
	17	4 wire plug connector pin (female)	Digikey	3M156151-ND	6	3.5	21	
	18	Power Barrel Connector	Digikey	CP-011B-ND	2	1.6	3.2	
	19	Cable assembly (to aux power)	Digikey	CP-2188-ND	2	2.5	5	

	20	USB Type B - Type A Adapter	Allied Electronics	70126196	1	10.05	10.05	
	21	USB 2.0 Cable (A Male to B Male)	Digikey	1175-1088-ND	1	3.25	3.25	
	22	Toggle Switch (DPDT)	Digikey	EG2398-ND	1	3.25	3.25	
	23	2-pin JST SM Plug + Receptacle Cable Set	Adafruit	2880	6	0.75	4.5	
	24	Peltier Plate	Digikey	926-1267-ND	6	65.92	395.52	
	25	CPU Heat Sink	Newegg	N82E16835114039	6	25.75	154.5	
	26	Surface Thermistor (10kohm)	Omega	SA2F-TH-44031-40	6	68.5	411	
	27	Precision Resistor (5kohm)	Digikey	696-1566-ND	6	6.12	36.72	
	28	Copper Plate	McMaster	8963K703	1	45.19	45.19	needs to be machined (see CAD file)
	29	HDPE Plastic Casing	McMaster	8619K482	1	34.31	34.31	needs to be machined (see CAD file)
	30	Aluminum Plate	McMaster	8975K437	1	34.8	34.8	needs to be machined (see CAD file)
	31	Foam Insulation	McMaster	5541K11	1	10.62	10.62	
	32	Plastic screws	McMaster	94323A837	1	9.93	9.93	
	33	Plastic legs	McMaster	8624K12	1	6.72	6.72	needs to be machined (see CAD file)
	34	Rubber feet	McMaster	9541K2	1	12.48	12.48	
	35	Aluminum bracket	McMaster	7062T13	1	7.9	7.9	needs to be machined (see CAD file)
	36	Bracket rubber seal	McMaster	1129A989	1	8.75	8.75	
	37	18-8 Screws	McMaster	92196A540	1	9.53	9.53	
	38	Silicon Wafer	University Wafer	857	6	23.9	143.4	
	39	Thermal Paste	Amazon	AS5-3.5G	1	6	6	
Pressure Delivery System	1	Valve (set of 4) actuator	KSCDirect	ASC 18800056	0.5	278.75	139.375	
	2	Valve (set of 2) actuator	KSCDirect	ASC 18800054	0.5	145.02	72.51	
	3	Valve Connector	KSCDirect	ASC 88118802	1	11.3	11.3	
	4	Project Box (for Pressure Box) (11.5"x7.5"x7")	Allied Electronics	70164768	1	103.45	103.45	needs to be machined
	5	USB Type B - Type A Adapter	Allied Electronics	70126196	2	10.05	20.1	
	6	USB 2.0 Cable (A Male to B Male)	Digikey	1175-1088-ND	2	3.25	6.5	

	7	USB 2.0 Cable (A Male to B Male)	Digikey	1597-1533-ND	2	1.99	3.98	
	8	PacDrive USB driver board Type: Special (Outputs OFF at power on)	Ultimarc	PPACDSP+LU04	1	30	30	
	9	Regulator (0.5-60 PSI) for valves	McMaster	43275K14	3	40	120	
	10	Pressure gauge (0-60 PSI) for valves	McMaster	3846K43	1	12.39	12.39	
	11	Pressure gauge (0-15 PSI) for valves	McMaster	3846K41	2	10.46	20.92	
	12	Polybutylene & Brass Push-to-Connect Fitting Adapter from pressure gauges to push to connect fitting	McMaster	5111K664	3	3.57	10.71	
	13	Polybutylene & Nickel-Pltd Brass adapter from pressure regulators and valve manifold to push to connect fitting	McMaster	52065K124	6	2.35	14.1	
	14	Y airway splitter	McMaster	5111K402	2	5.1	10.2	
	15	Miniature Brass Coupler to external outlets	McMaster	5454K85	1	10.31	10.31	
	16	Polybutylene & Brass Push-to-Connect Fitting Tee airway splitter	McMaster	5111K172	3	3.4	10.2	
	17	Nylon and Nickel-Plated Brass Tube Fitting Adapter for couples valve manifold to external outlets	McMaster	5779K244	6	2.76	16.56	
	18	Polybutylene & Brass panel mount coupler to external pressure source	McMaster	5111K202	1	9.3	9.3	
	19	Polybutylene & Brass adapter to external pressure source	McMaster	5111K92	1	1.89	1.89	
	20	Nylon tubing for internal air connections	McMaster	5112K52	2	0.31	0.62	
	21	Male Luer Integral Lock Ring to 10-32 Special Tapered Thread, Natural Polypropylene	Nordson Medical	XMTLL-6	12	0.3498	4.1976	

	22	Power supply	DigiKey	271-2592-ND	1	23.41	23.41	
	23	Panel mount plugs	DigiKey	CP-011B-ND	10	1.44	14.4	
	24	Power switch	DigiKey	679-1211-ND	1	7.22	7.22	
	25	Protection diode 1A	Digikey	1N4004RLGO SCT-ND	10	1.87	18.7	
	26	Panel mount LED	Digikey	350-2101-ND	1	5.24	5.24	
	27	Screw terminal, connects wires	DigiKey	WM15900-ND	5	1.79	8.95	
	28	Cable assumbly (to aux power)	Digikey	CP-2188-ND	2	2.5	5	
	29	Solenoid pinch valve (2-way normally closed)	Cole Parmer	EW-98302-10	6	99.5	597	
	30	4-40 Machine Screws	Allied Electronics	70126097	0.1	8.92	0.892	
Clog Detection System	1	Arduino MEGA	Arduino	A000067	1	38.5	38.5	
	2	4 wire plug connector pin (male)	Digikey	3M155973-ND	6	3.22	19.32	
	3	4 wire plug connector pin (female)	Digikey	3M156151-ND	6	3.5	21	
	4	Pressure sensors (15 PSI)	Mouser	785-24PCCFG6G	6	41.65	249.9	
	5	5V voltage regulator	Mouser	511-L7805CV	1	10.05	10.05	
	6	510ohm resistor	Mouser	667-ERG-1SJ511	6	3.25	19.5	
	7	Instrumentation amplifier	Mouser	595-INA126PA	6	3.15	18.9	
	8	3 wire plug connector pin (male)	Digikey	3M155968-ND	1	2.72	2.72	
	9	3 wire plug connector pin (female)	Digikey	3M155845-ND	1	2.92	2.92	
	10	Solder Prototyping Breadboard	Digikey	SBB206-ND	1	2.49	2.49	
Misc. Items	1	Multi-position magnetic stirrers	VWR	12621-046	1	1609	1609	
	2	Power supply	Newegg	N82E1681743 8018	1	119.99	119.99	
	3	Cable set	Newegg	9SIAA6P4G38 262	1	49.9	49.9	
	4	Heat shrink tubing	Amazon	B075WR9FVL	1	7.99	7.99	
	5	Wire	Digikey	C2016B-100-ND	1	18.7	18.7	

Supplementary Note 2. LASSO regression

Below is a list of behavioral metrics that were extracted from the data set. This is by no means an exhaustive list of potential extracted behavioral metrics, but simply what we used to perform LASSO regression to observe how stereotyped lifespan is across a population in relation to behavioral decline over time. Bolded variables indicated variables actually inputted into the LASSO regression model for the wild-type population cultured at 25°C with food levels of OD_{600}5. Other variables were censored due to data sparsity. Additionally, Day 0 indicates activity at the L4 larval stage.

'ampData_maxVal_maxValDay_Day0'
'ampData_maxVal_maxValDay_Day1'
'ampData_maxVal_maxValDay_Day2'
'ampData_maxVal_maxValDay_Day3'
'ampData_maxVal_maxValDay_Day4'
'ampData_maxVal_maxValDay_Day5'
'ampData_maxVal_maxValDay_Day6'
'ampData_maxVal_maxValDay_Day7'
'ampData_maxVal_maxValDay_Day8'
'ampData_maxVal_maxValDay_Day9'
'ampData_maxVal_maxValDay_Day10'
'ampData_maxVal_avgValDay_Day0'
'ampData_maxVal_avgValDay_Day1'
'ampData_maxVal_avgValDay_Day2'
'ampData_maxVal_avgValDay_Day3'
'ampData_maxVal_avgValDay_Day4'
'ampData_maxVal_avgValDay_Day5'
'ampData_maxVal_avgValDay_Day6'
'ampData_maxVal_avgValDay_Day7'
'ampData_maxVal_avgValDay_Day8'
'ampData_maxVal_avgValDay_Day9'
'ampData_maxVal_avgValDay_Day10'
'ampData_maxVal_maxValDay_high_Day0'
'ampData_maxVal_maxValDay_high_Day1'
'ampData_maxVal_maxValDay_high_Day2'
'ampData_maxVal_maxValDay_high_Day3'
'ampData_maxVal_maxValDay_high_Day4'
'ampData_maxVal_maxValDay_high_Day5'
'ampData_maxVal_maxValDay_high_Day6'
'ampData_maxVal_maxValDay_high_Day7'
'ampData_maxVal_maxValDay_high_Day8'
'ampData_maxVal_maxValDay_high_Day9'
'ampData_maxVal_maxValDay_high_Day10'
'ampData_maxVal_avgValDay_high_Day0'
'ampData_maxVal_avgValDay_high_Day1'

'csData_maxVal_maxValDay_Day0'
'csData_maxVal_maxValDay_Day1'
'csData_maxVal_maxValDay_Day2'
'csData_maxVal_maxValDay_Day3'
'csData_maxVal_maxValDay_Day4'
'csData_maxVal_maxValDay_Day5'
'csData_maxVal_maxValDay_Day6'
'csData_maxVal_maxValDay_Day7'
'csData_maxVal_maxValDay_Day8'
'csData_maxVal_maxValDay_Day9'
'csData_maxVal_maxValDay_Day10'
'csData_maxVal_avgValDay_Day0'
'csData_maxVal_avgValDay_Day1'
'csData_maxVal_avgValDay_Day2'
'csData_maxVal_avgValDay_Day3'
'csData_maxVal_avgValDay_Day4'
'csData_maxVal_avgValDay_Day5'
'csData_maxVal_avgValDay_Day6'
'csData_maxVal_avgValDay_Day7'
'csData_maxVal_avgValDay_Day8'
'csData_maxVal_avgValDay_Day9'
'csData_maxVal_avgValDay_Day10'
'csData_maxVal_maxValDay_high_Day0'
'csData_maxVal_maxValDay_high_Day1'
'csData_maxVal_maxValDay_high_Day2'
'csData_maxVal_maxValDay_high_Day3'
'csData_maxVal_maxValDay_high_Day4'
'csData_maxVal_maxValDay_high_Day5'
'csData_maxVal_maxValDay_high_Day6'
'csData_maxVal_maxValDay_high_Day7'
'csData_maxVal_maxValDay_high_Day8'
'csData_maxVal_maxValDay_high_Day9'
'csData_maxVal_maxValDay_high_Day10'
'csData_maxVal_avgValDay_high_Day0'
'csData_maxVal_avgValDay_high_Day1'

'ampData_maxVal_avgValDay_high_Day2'
'ampData_maxVal_avgValDay_high_Day3'
'ampData_maxVal_avgValDay_high_Day4'
'ampData_maxVal_avgValDay_high_Day5'
'ampData_maxVal_avgValDay_high_Day6'
'ampData_maxVal_avgValDay_high_Day7'
'ampData_maxVal_avgValDay_high_Day8'
'ampData_maxVal_avgValDay_high_Day9'
'ampData_maxVal_avgValDay_high_Day10'
'ampData_maxVal_declinePT_duration'
'ampData_maxVal_relativeHighPeriod_duration'
'ampData_meanVal_maxValDay_Day0'
'ampData_meanVal_maxValDay_Day1'
'ampData_meanVal_maxValDay_Day2'
'ampData_meanVal_maxValDay_Day3'
'ampData_meanVal_maxValDay_Day4'
'ampData_meanVal_maxValDay_Day5'
'ampData_meanVal_maxValDay_Day6'
'ampData_meanVal_maxValDay_Day7'
'ampData_meanVal_maxValDay_Day8'
'ampData_meanVal_maxValDay_Day9'
'ampData_meanVal_maxValDay_Day10'
'ampData_meanVal_avgValDay_Day0'
'ampData_meanVal_avgValDay_Day1'
'ampData_meanVal_avgValDay_Day2'
'ampData_meanVal_avgValDay_Day3'
'ampData_meanVal_avgValDay_Day4'
'ampData_meanVal_avgValDay_Day5'
'ampData_meanVal_avgValDay_Day6'
'ampData_meanVal_avgValDay_Day7'
'ampData_meanVal_avgValDay_Day8'
'ampData_meanVal_avgValDay_Day9'
'ampData_meanVal_avgValDay_Day10'
'ampData_meanVal_maxValDay_high_Day0'
'ampData_meanVal_maxValDay_high_Day1'
'ampData_meanVal_maxValDay_high_Day2'
'ampData_meanVal_maxValDay_high_Day3'
'ampData_meanVal_maxValDay_high_Day4'
'ampData_meanVal_maxValDay_high_Day5'
'ampData_meanVal_maxValDay_high_Day6'
'ampData_meanVal_maxValDay_high_Day7'
'ampData_meanVal_maxValDay_high_Day8'
'ampData_meanVal_maxValDay_high_Day9'

'csData_maxVal_avgValDay_high_Day2'
'csData_maxVal_avgValDay_high_Day3'
'csData_maxVal_avgValDay_high_Day4'
'csData_maxVal_avgValDay_high_Day5'
'csData_maxVal_avgValDay_high_Day6'
'csData_maxVal_avgValDay_high_Day7'
'csData_maxVal_avgValDay_high_Day8'
'csData_maxVal_avgValDay_high_Day9'
'csData_maxVal_avgValDay_high_Day10'
'csData_maxVal_declinePT_duration'
'csData_maxVal_relativeHighPeriod_duration'
'csData_meanVal_maxValDay_Day0'
'csData_meanVal_maxValDay_Day1'
'csData_meanVal_maxValDay_Day2'
'csData_meanVal_maxValDay_Day3'
'csData_meanVal_maxValDay_Day4'
'csData_meanVal_maxValDay_Day5'
'csData_meanVal_maxValDay_Day6'
'csData_meanVal_maxValDay_Day7'
'csData_meanVal_maxValDay_Day8'
'csData_meanVal_maxValDay_Day9'
'csData_meanVal_maxValDay_Day10'
'csData_meanVal_avgValDay_Day0'
'csData_meanVal_avgValDay_Day1'
'csData_meanVal_avgValDay_Day2'
'csData_meanVal_avgValDay_Day3'
'csData_meanVal_avgValDay_Day4'
'csData_meanVal_avgValDay_Day5'
'csData_meanVal_avgValDay_Day6'
'csData_meanVal_avgValDay_Day7'
'csData_meanVal_avgValDay_Day8'
'csData_meanVal_avgValDay_Day9'
'csData_meanVal_avgValDay_Day10'
'csData_meanVal_maxValDay_high_Day0'
'csData_meanVal_maxValDay_high_Day1'
'csData_meanVal_maxValDay_high_Day2'
'csData_meanVal_maxValDay_high_Day3'
'csData_meanVal_maxValDay_high_Day4'
'csData_meanVal_maxValDay_high_Day5'
'csData_meanVal_maxValDay_high_Day6'
'csData_meanVal_maxValDay_high_Day7'
'csData_meanVal_maxValDay_high_Day8'
'csData_meanVal_maxValDay_high_Day9'

'ampData_meanVal_maxValDay_high_Day10'
'ampData_meanVal_avgValDay_high_Day0'
'ampData_meanVal_avgValDay_high_Day1'
'ampData_meanVal_avgValDay_high_Day2'
'ampData_meanVal_avgValDay_high_Day3'
'ampData_meanVal_avgValDay_high_Day4'
'ampData_meanVal_avgValDay_high_Day5'
'ampData_meanVal_avgValDay_high_Day6'
'ampData_meanVal_avgValDay_high_Day7'
'ampData_meanVal_avgValDay_high_Day8'
'ampData_meanVal_avgValDay_high_Day9'
'ampData_meanVal_avgValDay_high_Day10'
'ampData_meanVal_declinePT_duration'
'ampData_meanVal_relativeHighPeriod_duration'
'dpData_maxVal_maxValDay_Day0'
'dpData_maxVal_maxValDay_Day1'
'dpData_maxVal_maxValDay_Day2'
'dpData_maxVal_maxValDay_Day3'
'dpData_maxVal_maxValDay_Day4'
'dpData_maxVal_maxValDay_Day5'
'dpData_maxVal_maxValDay_Day6'
'dpData_maxVal_maxValDay_Day7'
'dpData_maxVal_maxValDay_Day8'
'dpData_maxVal_maxValDay_Day9'
'dpData_maxVal_maxValDay_Day10'
'dpData_maxVal_avgValDay_Day0'
'dpData_maxVal_avgValDay_Day1'
'dpData_maxVal_avgValDay_Day2'
'dpData_maxVal_avgValDay_Day3'
'dpData_maxVal_avgValDay_Day4'
'dpData_maxVal_avgValDay_Day5'
'dpData_maxVal_avgValDay_Day6'
'dpData_maxVal_avgValDay_Day7'
'dpData_maxVal_avgValDay_Day8'
'dpData_maxVal_avgValDay_Day9'
'dpData_maxVal_avgValDay_Day10'
'dpData_maxVal_maxValDay_high_Day0'
'dpData_maxVal_maxValDay_high_Day1'
'dpData_maxVal_maxValDay_high_Day2'
'dpData_maxVal_maxValDay_high_Day3'
'dpData_maxVal_maxValDay_high_Day4'
'dpData_maxVal_maxValDay_high_Day5'
'dpData_maxVal_maxValDay_high_Day6'

'csData_meanVal_maxValDay_high_Day10'
'csData_meanVal_avgValDay_high_Day0'
'csData_meanVal_avgValDay_high_Day1'
'csData_meanVal_avgValDay_high_Day2'
'csData_meanVal_avgValDay_high_Day3'
'csData_meanVal_avgValDay_high_Day4'
'csData_meanVal_avgValDay_high_Day5'
'csData_meanVal_avgValDay_high_Day6'
'csData_meanVal_avgValDay_high_Day7'
'csData_meanVal_avgValDay_high_Day8'
'csData_meanVal_avgValDay_high_Day9'
'csData_meanVal_avgValDay_high_Day10'
'csData_meanVal_declinePT_duration'
'csData_meanVal_relativeHighPeriod_duration'
'freqData_meanVal_maxValDay_Day0'
'freqData_meanVal_maxValDay_Day1'
'freqData_meanVal_maxValDay_Day2'
'freqData_meanVal_maxValDay_Day3'
'freqData_meanVal_maxValDay_Day4'
'freqData_meanVal_maxValDay_Day5'
'freqData_meanVal_maxValDay_Day6'
'freqData_meanVal_maxValDay_Day7'
'freqData_meanVal_maxValDay_Day8'
'freqData_meanVal_maxValDay_Day9'
'freqData_meanVal_maxValDay_Day10'
'freqData_meanVal_avgValDay_Day0'
'freqData_meanVal_avgValDay_Day1'
'freqData_meanVal_avgValDay_Day2'
'freqData_meanVal_avgValDay_Day3'
'freqData_meanVal_avgValDay_Day4'
'freqData_meanVal_avgValDay_Day5'
'freqData_meanVal_avgValDay_Day6'
'freqData_meanVal_avgValDay_Day7'
'freqData_meanVal_avgValDay_Day8'
'freqData_meanVal_avgValDay_Day9'
'freqData_meanVal_avgValDay_Day10'
'freqData_meanVal_maxValDay_high_Day0'
'freqData_meanVal_maxValDay_high_Day1'
'freqData_meanVal_maxValDay_high_Day2'
'freqData_meanVal_maxValDay_high_Day3'
'freqData_meanVal_maxValDay_high_Day4'
'freqData_meanVal_maxValDay_high_Day5'
'freqData_meanVal_maxValDay_high_Day6'

'dpData_maxVal_maxValDay_high_Day7'
'dpData_maxVal_maxValDay_high_Day8'
'dpData_maxVal_maxValDay_high_Day9'
'dpData_maxVal_maxValDay_high_Day10'
'dpData_maxVal_avgValDay_high_Day0'
'dpData_maxVal_avgValDay_high_Day1'
'dpData_maxVal_avgValDay_high_Day2'
'dpData_maxVal_avgValDay_high_Day3'
'dpData_maxVal_avgValDay_high_Day4'
'dpData_maxVal_avgValDay_high_Day5'
'dpData_maxVal_avgValDay_high_Day6'
'dpData_maxVal_avgValDay_high_Day7'
'dpData_maxVal_avgValDay_high_Day8'
'dpData_maxVal_avgValDay_high_Day9'
'dpData_maxVal_avgValDay_high_Day10'
'dpData_maxVal_declinePT_duration'
'dpData_maxVal_relativeHighPeriod_duration'
'dpData_meanVal_maxValDay_Day0'
'dpData_meanVal_maxValDay_Day1'
'dpData_meanVal_maxValDay_Day2'
'dpData_meanVal_maxValDay_Day3'
'dpData_meanVal_maxValDay_Day4'
'dpData_meanVal_maxValDay_Day5'
'dpData_meanVal_maxValDay_Day6'
'dpData_meanVal_maxValDay_Day7'
'dpData_meanVal_maxValDay_Day8'
'dpData_meanVal_maxValDay_Day9'
'dpData_meanVal_maxValDay_Day10'
'dpData_meanVal_avgValDay_Day0'
'dpData_meanVal_avgValDay_Day1'
'dpData_meanVal_avgValDay_Day2'
'dpData_meanVal_avgValDay_Day3'
'dpData_meanVal_avgValDay_Day4'
'dpData_meanVal_avgValDay_Day5'
'dpData_meanVal_avgValDay_Day6'
'dpData_meanVal_avgValDay_Day7'
'dpData_meanVal_avgValDay_Day8'
'dpData_meanVal_avgValDay_Day9'
'dpData_meanVal_avgValDay_Day10'
'dpData_meanVal_maxValDay_high_Day0'
'dpData_meanVal_maxValDay_high_Day1'
'dpData_meanVal_maxValDay_high_Day2'
'dpData_meanVal_maxValDay_high_Day3'

'freqData_meanVal_maxValDay_high_Day7'
'freqData_meanVal_maxValDay_high_Day8'
'freqData_meanVal_maxValDay_high_Day9'
'freqData_meanVal_maxValDay_high_Day10'
'freqData_meanVal_avgValDay_high_Day0'
'freqData_meanVal_avgValDay_high_Day1'
'freqData_meanVal_avgValDay_high_Day2'
'freqData_meanVal_avgValDay_high_Day3'
'freqData_meanVal_avgValDay_high_Day4'
'freqData_meanVal_avgValDay_high_Day5'
'freqData_meanVal_avgValDay_high_Day6'
'freqData_meanVal_avgValDay_high_Day7'
'freqData_meanVal_avgValDay_high_Day8'
'freqData_meanVal_avgValDay_high_Day9'
'freqData_meanVal_avgValDay_high_Day10'
'freqData_meanVal_declinePT_duration'
'freqData_meanVal_relativeHighPeriod_duration'
'freqData_maxVal_maxValDay_Day0'
'freqData_maxVal_maxValDay_Day1'
'freqData_maxVal_maxValDay_Day2'
'freqData_maxVal_maxValDay_Day3'
'freqData_maxVal_maxValDay_Day4'
'freqData_maxVal_maxValDay_Day5'
'freqData_maxVal_maxValDay_Day6'
'freqData_maxVal_maxValDay_Day7'
'freqData_maxVal_maxValDay_Day8'
'freqData_maxVal_maxValDay_Day9'
'freqData_maxVal_maxValDay_Day10'
'freqData_maxVal_avgValDay_Day0'
'freqData_maxVal_avgValDay_Day1'
'freqData_maxVal_avgValDay_Day2'
'freqData_maxVal_avgValDay_Day3'
'freqData_maxVal_avgValDay_Day4'
'freqData_maxVal_avgValDay_Day5'
'freqData_maxVal_avgValDay_Day6'
'freqData_maxVal_avgValDay_Day7'
'freqData_maxVal_avgValDay_Day8'
'freqData_maxVal_avgValDay_Day9'
'freqData_maxVal_avgValDay_Day10'
'freqData_maxVal_maxValDay_high_Day0'
'freqData_maxVal_maxValDay_high_Day1'
'freqData_maxVal_maxValDay_high_Day2'
'freqData_maxVal_maxValDay_high_Day3'

'dpData_meanVal_maxValDay_high_Day4'
'dpData_meanVal_maxValDay_high_Day5'
'dpData_meanVal_maxValDay_high_Day6'
'dpData_meanVal_maxValDay_high_Day7'
'dpData_meanVal_maxValDay_high_Day8'
'dpData_meanVal_maxValDay_high_Day9'
'dpData_meanVal_maxValDay_high_Day10'
'dpData_meanVal_avgValDay_high_Day0'
'dpData_meanVal_avgValDay_high_Day1'
'dpData_meanVal_avgValDay_high_Day2'
'dpData_meanVal_avgValDay_high_Day3'
'dpData_meanVal_avgValDay_high_Day4'
'dpData_meanVal_avgValDay_high_Day5'
'dpData_meanVal_avgValDay_high_Day6'
'dpData_meanVal_avgValDay_high_Day7'
'dpData_meanVal_avgValDay_high_Day8'
'dpData_meanVal_avgValDay_high_Day9'
'dpData_meanVal_avgValDay_high_Day10'
'dpData_meanVal_declinePT_duration'
'dpData_meanVal_relativeHighPeriod_duration'
'PCData_maxValDay_Day0'
'PCData_maxValDay_Day1'
'PCData_maxValDay_Day2'
'PCData_maxValDay_Day3'
'PCData_maxValDay_Day4'
'PCData_maxValDay_Day5'
'PCData_maxValDay_Day6'
'PCData_maxValDay_Day7'
'PCData_maxValDay_Day8'
'PCData_maxValDay_Day9'
'PCData_maxValDay_Day10'
'PCData_avgValDay_Day0'
'PCData_avgValDay_Day1'
'PCData_avgValDay_Day2'
'PCData_avgValDay_Day3'
'PCData_avgValDay_Day4'
'PCData_avgValDay_Day5'
'PCData_avgValDay_Day6'
'PCData_avgValDay_Day7'
'PCData_avgValDay_Day8'
'PCData_avgValDay_Day9'
'PCData_avgValDay_Day10'
'PCData_maxValDay_high_Day0'

'freqData_maxVal_maxValDay_high_Day4'
'freqData_maxVal_maxValDay_high_Day5'
'freqData_maxVal_maxValDay_high_Day6'
'freqData_maxVal_maxValDay_high_Day7'
'freqData_maxVal_maxValDay_high_Day8'
'freqData_maxVal_maxValDay_high_Day9'
'freqData_maxVal_maxValDay_high_Day10'
'freqData_maxVal_avgValDay_high_Day0'
'freqData_maxVal_avgValDay_high_Day1'
'freqData_maxVal_avgValDay_high_Day2'
'freqData_maxVal_avgValDay_high_Day3'
'freqData_maxVal_avgValDay_high_Day4'
'freqData_maxVal_avgValDay_high_Day5'
'freqData_maxVal_avgValDay_high_Day6'
'freqData_maxVal_avgValDay_high_Day7'
'freqData_maxVal_avgValDay_high_Day8'
'freqData_maxVal_avgValDay_high_Day9'
'freqData_maxVal_avgValDay_high_Day10'
'freqData_maxVal_declinePT_duration'
'freqData_maxVal_relativeHighPeriod_duration'
'freqData_numSeg_maxValDay_Day0'
'freqData_numSeg_maxValDay_Day1'
'freqData_numSeg_maxValDay_Day2'
'freqData_numSeg_maxValDay_Day3'
'freqData_numSeg_maxValDay_Day4'
'freqData_numSeg_maxValDay_Day5'
'freqData_numSeg_maxValDay_Day6'
'freqData_numSeg_maxValDay_Day7'
'freqData_numSeg_maxValDay_Day8'
'freqData_numSeg_maxValDay_Day9'
'freqData_numSeg_maxValDay_Day10'
'freqData_numSeg_avgValDay_Day0'
'freqData_numSeg_avgValDay_Day1'
'freqData_numSeg_avgValDay_Day2'
'freqData_numSeg_avgValDay_Day3'
'freqData_numSeg_avgValDay_Day4'
'freqData_numSeg_avgValDay_Day5'
'freqData_numSeg_avgValDay_Day6'
'freqData_numSeg_avgValDay_Day7'
'freqData_numSeg_avgValDay_Day8'
'freqData_numSeg_avgValDay_Day9'
'freqData_numSeg_avgValDay_Day10'
'freqData_numSeg_maxValDay_high_Day0'

'PCData_maxValDay_high_Day1'
'PCData_maxValDay_high_Day2'
'PCData_maxValDay_high_Day3'
'PCData_maxValDay_high_Day4'
'PCData_maxValDay_high_Day5'
'PCData_maxValDay_high_Day6'
'PCData_maxValDay_high_Day7'
'PCData_maxValDay_high_Day8'
'PCData_maxValDay_high_Day9'
'PCData_maxValDay_high_Day10'
'PCData_avgValDay_high_Day0'
'PCData_avgValDay_high_Day1'
'PCData_avgValDay_high_Day2'
'PCData_avgValDay_high_Day3'
'PCData_avgValDay_high_Day4'
'PCData_avgValDay_high_Day5'
'PCData_avgValDay_high_Day6'
'PCData_avgValDay_high_Day7'
'PCData_avgValDay_high_Day8'
'PCData_avgValDay_high_Day9'
'PCData_avgValDay_high_Day10'
'PCData_declinePT_duration'
'PCData_relativeHighPeriod_duration'
'freqData_numSeg_maxValDay_high_Day1'
'freqData_numSeg_maxValDay_high_Day2'
'freqData_numSeg_maxValDay_high_Day3'
'freqData_numSeg_maxValDay_high_Day4'
'freqData_numSeg_maxValDay_high_Day5'
'freqData_numSeg_maxValDay_high_Day6'
'freqData_numSeg_maxValDay_high_Day7'
'freqData_numSeg_maxValDay_high_Day8'
'freqData_numSeg_maxValDay_high_Day9'
'freqData_numSeg_maxValDay_high_Day10'
'freqData_numSeg_avgValDay_high_Day0'
'freqData_numSeg_avgValDay_high_Day1'
'freqData_numSeg_avgValDay_high_Day2'
'freqData_numSeg_avgValDay_high_Day3'
'freqData_numSeg_avgValDay_high_Day4'
'freqData_numSeg_avgValDay_high_Day5'
'freqData_numSeg_avgValDay_high_Day6'
'freqData_numSeg_avgValDay_high_Day7'
'freqData_numSeg_avgValDay_high_Day8'
'freqData_numSeg_avgValDay_high_Day9'
'freqData_numSeg_avgValDay_high_Day10'
'freqData_numSeg_declinePT_duration'
'freqData_numSeg_relativeHighPeriod_duration'

Below is a table of the selected variables for the LASSO model for the wild-type population cultured at 25°C at a food level of OD_{6005}, along with the corresponding least-squares regression coefficient values.

Behavioral Metric	Description	LSR Coefficient
'ampData_maxVal_avgValDay_Day0'	Average amplitude of L4 stage	0.040235
'ampData_maxVal_maxValDay_high_Day7'	Maximum amplitude of high movement activity of Day 7 adult	-0.00035
'ampData_maxVal_avgValDay_high_Day6'	Average amplitude of high movement activity of Day 6 adult	0.011228
'ampData_maxVal_avgValDay_high_Day8'	Average amplitude of high movement activity of Day 8 adult	0.026329
'csData_maxVal_avgValDay_Day1'	Average centroid speed of Day 1 adult	0.007468
'csData_maxVal_maxValDay_high_Day1'	Maximum centroid speed of high movement activity of Day 1 adult	0.024184

'csData_maxVal_declinePT_duration'	Duration of high activity centroid speed movement	0.199274
'dpData_maxVal_relativeHighPeriod_duration'	Normalized duration of high activity of maximum pixel difference across frames	-0.39457
'freqData_numSeg_avgValDay_high_Day8'	Average number of body segments undergoing swimming of high movement activity of Day 8 adult	0.027288
'PCData_avgValDay_Day8'	Average raw movement of Day 8 adult	0.047573
'PCData_avgValDay_Day9'	Average raw movement of Day 9 adult	0.053078
'PCData_declinePT_duration'	Duration of high activity of raw movement	3.66405
'PCData_relativeHighPeriod_duration'	Normalized duration of high activity of raw movement	-3.44935

Supplementary References

1. Lucanic, M., et al., *Impact of genetic background and experimental reproducibility on identifying chemical compounds with robust longevity effects.* Nature Communications, 2017. **8**: p. 14256.
2. Chung, K., et al., *Microfluidic chamber arrays for whole-organism behavior-based chemical screening.* Lab on a Chip, 2011. **11**: p. 3689-3697.
