# Supplementary material for: An automated platform to monitor long-term behavior and healthspan in Caenorhabditis elegans under precise environmental control
Source: Commun Biol. 2020 Jun 10;3:297. doi: 10.1038/s42003-020-1013-2 (PMC7287092; doi:10.1038/s42003-020-1013-2)
Supplement: Supplementary file 20 — Description of Additional Supplementary Files [file 42003_2020_1013_MOESM20_ESM.docx]

**Supplementary Movies 1-3.** Time-lapse videos of a wild-type worm in device cultured at 25°C at OD_600_5

Compiled time-lapse videos of individuals cultured on HeALTH from the initial L4 stage to the end of the lifespan. The worms are wild-type (N2) and are cultured at 25°C at OD_600_5 from Day 2 adult to death. Each time-lapse video tracks a separate worm. From L4 larval stage to Day 1 adult the worms were cultured in OD_600_10 and at 20°C to ensure no adverse developmental effects from low food levels or increased temperature. The videos are shown at twice the speed.

**Supplementary Movies 4-6.** Time-lapse videos of a wild-type worm in device cultured at 25°C at OD_600_10

Compiled time-lapse videos of individuals cultured on HeALTH from the initial L4 stage to the end of the lifespan. The worms are wild-type (N2) and are cultured at 25°C at OD_600_10 from Day 2 adult to death. Each time-lapse video tracks a separate worm. From L4 larval stage to Day 1 adult the worms were cultured in OD_600_10 and at 20°C to ensure no adverse developmental effects from low food levels or increased temperature. The videos are shown at twice the speed.

**Supplementary Movies 7-9.** Time-lapse videos of a wild-type worm in device cultured at 25°C at OD_600_2.5

Compiled time-lapse videos of individuals cultured on HeALTH from the initial L4 stage to the end of the lifespan. The worms are wild-type (N2) and are cultured at 25°C at OD_600_2.5 from Day 2 adult to death. Each time-lapse video tracks a separate worm. From L4 larval stage to Day 1 adult the worms were cultured in OD_600_10 and at 20°C to ensure no adverse developmental effects from low food levels or increased temperature. The videos are shown at twice the speed.

**Supplementary Movies 10-12.** Time-lapse videos of a *daf-16* worm in device cultured at 25°C at OD_600_5

Compiled time-lapse videos of individuals cultured on HeALTH from the initial L4 stage to the end of the lifespan. The worms are *daf-16* worm and are cultured at 25°C at OD_600_5 from Day 2 adult to death. Each time-lapse video tracks a separate worm. From L4 larval stage to Day 1 adult the worms were cultured in OD_600_10 and at 20°C to ensure no adverse developmental effects from low food levels or increased temperature. The videos are shown at twice the speed.

**Supplementary Movies 13-15.** Time-lapse videos of a *daf-2* worm in device cultured at 25°C at OD_600_5

Compiled time-lapse videos of individuals cultured on HeALTH from the initial L4 stage to the end of the lifespan. The worms are *daf-2* and are cultured at 25°C at OD_600_5 from Day 2 adult to death. Each time-lapse video tracks a separate worm. From L4 larval stage to Day 1 adult the worms were cultured in OD_600_10 and at 20°C to ensure no adverse developmental effects from low food levels or increased temperature. The videos are shown at twice the speed.

**Supplementary Data 1**

Raw lifespan data for the animals cultured in the HeALTH system and on traditional plate assays.

**Supplementary Data 2**

Raw behavioral metrics from the animals cultured in the HeALTH system.
