# Supplementary material for: An automated platform to monitor long-term behavior and healthspan in Caenorhabditis elegans under precise environmental control
Source: Commun Biol. 2020 Jun 10;3:297. doi: 10.1038/s42003-020-1013-2 (PMC7287092; doi:10.1038/s42003-020-1013-2)
Supplement: Supplementary file 21 — Reporting Summary [file 42003_2020_1013_MOESM21_ESM.pdf]

nature research

Corresponding author(s): Hang Lu

Last updated by author(s): May 5, 2020

Reporting Summary

Nature Research wishes to improve the reproducibility of the work that we publish. This form provides structure for consistency and transparency in reporting. For further information on Nature Research policies, see Authors & Referees and the Editorial Policy Checklist .

Statistics

For all statistical analyses, confirm that the following items are present in the figure legend, table legend, main text, or Methods section.

n/a	Confirmed	
☐	☒	The exact sample size (*n*) for each experimental group/condition, given as a discrete number and unit of measurement
☐	☒	A statement on whether measurements were taken from distinct samples or whether the same sample was measured repeatedly
☐	☒	The statistical test(s) used AND whether they are one- or two-sided<br>*Only common tests should be described solely by name; describe more complex techniques in the Methods section.*
☒	☐	A description of all covariates tested
☐	☒	A description of any assumptions or corrections, such as tests of normality and adjustment for multiple comparisons
☐	☒	A full description of the statistical parameters including central tendency (e.g. means) or other basic estimates (e.g. regression coefficient) AND variation (e.g. standard deviation) or associated estimates of uncertainty (e.g. confidence intervals)
☐	☒	For null hypothesis testing, the test statistic (e.g. *F*, *t*, *r*) with confidence intervals, effect sizes, degrees of freedom and *P* value noted<br>*Give P values as exact values whenever suitable.*
☒	☐	For Bayesian analysis, information on the choice of priors and Markov chain Monte Carlo settings
☒	☐	For hierarchical and complex designs, identification of the appropriate level for tests and full reporting of outcomes
☒	☐	Estimates of effect sizes (e.g. Cohen's *d*, Pearson's *r*), indicating how they were calculated

Our web collection on statistics for biologists contains articles on many of the points above.

Software and code

Policy information about availability of computer code

Data collection	A custom LabVIEW (2013) code was created to obtain and record the behavioral videos analyzed in this study and is deposited on GitHUB (https://github.com/kim-le63/HeALTH_Tracker/)
Data analysis	Behavioral analysis of the video data was performed with a custom MATLAB (2018b) script, which is deposited on GitHub (https://github.com/kim-le63/HeALTH_Tracker/). To characterize variance across trials, we used the lme4 v.1.12 package in R, which is freely available online. Survival analysis for longevity data was performed in the statistical analysis software JMP Pro14. Two-sample Kolmogorov-Smirnov tests were performed in MATLAB (2018b) using a built-in function. Remaining statistical tests (such as one-way ANOVA followed by Tukey's HSD test, Pearson's correlation coefficient) were performed in GraphPad Prism 5.

For manuscripts utilizing custom algorithms or software that are central to the research but not yet described in published literature, software must be made available to editors/reviewers. We strongly encourage code deposition in a community repository (e.g. GitHub). See the Nature Research guidelines for submitting code & software for further information.

Data

Policy information about availability of data

All manuscripts must include a data availability statement. This statement should provide the following information, where applicable:

- Accession codes, unique identifiers, or web links for publicly available datasets
- A list of figures that have associated raw data
- A description of any restrictions on data availability

Raw lifespan data for all individuals is included as a supplement. Behavioral information for each subpopulation is also included as a supplement. Raw behavioral videos will be available on request from the authors.

Field-specific reporting

Please select the one below that is the best fit for your research. If you are not sure, read the appropriate sections before making your selection.

☒ Life sciences ☐ Behavioural & social sciences ☐ Ecological, evolutionary & environmental sciences

For a reference copy of the document with all sections, see nature.com/documents/nr-reporting-summary-flat.pdf

Life sciences study design

All studies must disclose on these points even when the disclosure is negative.

Sample size	Many comparable biological experiments examining lifespan and healthspan metrics in C. elegans have sample sizes ranging from 20-100 individuals. To demonstrate a proof of concept, we aimed for sample sizes within the upper range of a tradition assay for each analyzed experimental condition (i.e. >75 individuals).
Data exclusions	Individuals were excluded from analysis due to multiple loadings within a chamber (thus making it impossible to track individual behavioral data over time due to lack of accurate tracking/identification over time). Individuals were also censored if they underwent premature forms of death not due to aging, such as bagging (death from internal hatching of eggs), rupturing/bursting (intestinal extrusion through the vulva), or if they crawled off the plate onto the wall (for the plate control assays). Videos were excluded if there were issues with recording (ex. frozen frames, issues with illumination, etc.). Behavioral data was excluded (i.e. designated as NaN) if segmentation could not be properly achieved in the video.
Replication	Each experimental condition had at least three biological replicates performed at different periods of time throughout the year. We characterized trial-to-trial variability for data gathered using our system and found that it was comparable to traditional methods.
Randomization	Individuals used for long-term culture and behavioral monitoring are randomly subsampled from a larger population. A small amount of buffer is added to a large plate with the entire population and is washed off to collect a random subpopulation for use. From this subpopulation, another random subsample is actually then loaded onto the microfluidic chip for monitoring. Microfluidic chips were then randomly chosen for experimental groups.
Blinding	Data collection was performed automatically, preventing human/experimental bias. To validate the accuracy of our automated live/dead code, the manual annotation was performed without knowing the identity or experimental conditions the worms were subjected to, in order to prevent bias in scoring. Subsequent analysis was done using an automated code, reducing the likelihood of experimental bias in scoring.

Reporting for specific materials, systems and methods

We require information from authors about some types of materials, experimental systems and methods used in many studies. Here, indicate whether each material, system or method listed is relevant to your study. If you are not sure if a list item applies to your research, read the appropriate section before selecting a response.

Materials & experimental systems

n/a	Involved in the study	
☒	☐	Antibodies
☒	☐	Eukaryotic cell lines
☒	☐	Palaeontology
☐	☒	Animals and other organisms
☒	☐	Human research participants
☒	☐	Clinical data

Methods

n/a	Involved in the study	
☒	☐	ChIP-seq
☒	☐	Flow cytometry
☒	☐	MRI-based neuroimaging

Animals and other organisms

Policy information about studies involving animals; ARRIVE guidelines recommended for reporting animal research

Laboratory animals	We use laboratory strains of Caenorhabditis elegans for our study. We use three different strains – N2 (the wild-type), daf-16 (mu86), and daf-2(e1368). All individuals were hermaphrodites and were initially at the L4 developmental stage at the start of the experiment.
Wild animals	No wild animals were used in this study.
Field-collected samples	No field-collected samples were used in the study.
Ethics oversight	No ethical approval or guidance was required in the No field-collected samples were used in the study.study due to the sole use of laboratory strains of C. elegans.

Note that full information on the approval of the study protocol must also be provided in the manuscript.
